# Impact of mtG3PDH inhibitors on proliferation and metabolism of androgen receptor-negative prostate cancer cells: Role of extracellular pyruvate

**DOI:** 10.1371/journal.pone.0325509

**Published:** 2025-06-09

**Authors:** Floriana Jessica Di Paola, Luiza HD Cardoso, Efterpi Nikitopoulou, Bianca Kulik, Sandra Rühl, Alexander Eva, Natascha Sommer, Thomas Linn, Erich Gnaiger, Klaus Failing, Kathrin Büttner, Christian Frezza, Sybille Mazurek

**Affiliations:** 1 Institute of Veterinary Physiology and Biochemistry, Justus Liebig University of Giessen, Giessen, Germany; 2 IRCCS Azienda Ospedaliero-Universitaria di Bologna, Bologna, Italy,; 3 Oroboros Instruments, Schoepfstrasse 18, Innsbruck, Austria,; 4 Medical Research Council Cancer Unit, University of Cambridge, Hutchison/MRC Research Centre, Cambridge Biomedical Campus, Cambridge, United Kingdom; 5 Excellence Cluster Cardio-Pulmonary Institute (CPI), University of Giessen and Marburg Lung Center (UGMLC), Member of the German Center for Lung Research (DZL), Justus Liebig University, Giessen, Germany; 6 Clinical Research Unit, Center of Internal Medicine, Justus Liebig University, Giessen, Germany; 7 Unit for Biomathematics and Data Processing, Veterinary Faculty, Justus Liebig University of Giessen, Giessen, Germany; 8 Faculty of Medicine and University Hospital Cologne, Faculty of Mathematics and Natural Sciences, Cluster of Excellence Cellular Stress Responses in Aging-associated Diseases (CECAD), University of Cologne, Cologne, Germany; Georgetown University Medical Centre, UNITED STATES OF AMERICA

## Abstract

Mitochondrial glycerol 3-P dehydrogenase (mtG3PDH) plays a significant role in cellular bioenergetics by serving as a rate-limiting element in the glycerophosphate shuttle, which connects cytosolic glycolysis to mitochondrial oxidative metabolism. mtG3PDH was identified as an important site of electron leakage leading to ROS production to the mitochondrial matrix and intermembrane space. Our research focused on the role of two published mtG3PDH inhibitors (RH02211 and iGP-1) on the proliferation and metabolism of PC-3 and DU145 prostate cancer cells characterized by different mtG3PDH activities. Since pyruvate as a substrate of lactate dehydrogenase (LDH) may represent an escape mechanism for the recycling of cytosolic NAD^+^ via the glycerophosphate shuttle, we investigated the effect of pyruvate on the mode of action of the mtG3PDH inhibitors. Extracellular pyruvate weakened the growth-inhibitory effects of RH02211 and iGP-1 in PC-3 cells but not in DU145 cells, which correlated with higher H-type LDH and lower mitochondrial glutamate-oxaloacetate transaminase in DU145 cells. In the pyruvate-low medium, the strength of inhibition was more pronounced in PC-3 cells, characterized by higher mtG3PDH activities compared to DU145 cells. Pyruvate conversion rates (production in pyruvate-low and consumption in pyruvate-high PC-3 cells) were not impaired by RH02211 and iGP-1, suggesting that the conversion of extracellular pyruvate to lactate was not the primary factor responsible for the weakening effect of extracellular pyruvate on the RH02211-induced inhibition of PC-3 proliferation. In pyruvate-high PC-3 cells, the intracellular glycerol-3-P and dihydroxyacetone-P concentrations were consistent with an inhibition of mtG3PDH. In contrast, in pyruvate-low cells, the concentrations of these metabolites suggested an activation of mtG3PDH in parallel with an impairment of cytosolic G3PDH by RH02211. Of all metabolic characterizations recorded in this study (fluxes, intracellular intermediates, O_2_ consumption and H_2_O_2_ production), the decrease in glutaminolysis correlated best with the RH02211-induced inhibition of proliferation in pyruvate-low and pyruvate-high PC-3 cells.

## Introduction

Mitochondrial glycerol-3-phosphate dehydrogenase (mtG3PDH, GPD2) is a nuclear-encoded protein located on the outer surface of the mitochondrial inner membrane and is thus a component of the respiratory Complex (CGpDH) of the membrane-electron transfer system (ETS). As part of the glycerol-3-P shuttle, mtG3PDH, together with cytosolic glycerol-3-P dehydrogenase (cG3PDH) is involved in transferring hydrogen from cytosolic NADH + H+ produced within the glyceraldehyde-3-P dehydrogenase (GAPDH) reaction into the mitochondrial electron transfer system to restore cytosolic NAD+ and maintain glycolysis. Glycerol-3-P, the substrate of mtG3PDH, is synthesized from glycolytic dihydroxyacetone phosphate (DHAP). High mtG3PDH activity is described for several prostate cancer cell lines compared to benign prostate epithelial cells. At the same time, the overexpression of the cG3PDH isoenzyme and high glycerol-3-P concentrations have been shown to suppress the proliferation of cancer cells in vitro [1–4]. mtG3PDH has been shown to participate in the production of mitochondrial reactive oxygen species (ROS) in both the mitochondrial matrix and the intermembrane space [5,6]. Accordingly, in prostate cancer cells, ROS production increases compared to benign prostate cells [1,2].

High ROS concentrations stabilize the α-subunit of the hypoxia-inducible factor 1 (HIF1), a key transcriptional regulator of several glycolytic enzymes, including pyruvate kinase isoenzyme M2 (M2-PK, PKM2) and lactate dehydrogenase (LDH) [7–9]. In proliferating and especially tumor cells, glycolysis is a crucial source of energy and building blocks, such as nucleic acids, phospholipids, and amino acids. Both functions of glycolysis are necessary metabolic prerequisites for cell division [10,11].

Besides the glycerol-3-P shuttle, two other important metabolic mechanisms that recycle glycolytic NAD+ are the LDH reaction and the malate-aspartate shuttle. LDH is a tetrameric protein composed of different combinations of the subunits M (skeletal muscle type = A-type) and H (heart muscle type = B-type) [12]. The resulting isoenzymes differ in their isoelectric points (IEPs) [13,14]. The H-type dominates in tissues with high oxygen consumption and respiration rates, whereas the M-type, characterized by a high affinity for its substrate, pyruvate, predominates in tissues with high glycolytic activity [15]. Cancer cells are characterized by a decrease in the H-type with simultaneous overexpression of the M-type of LDH [16,17]. The malate-aspartate shuttle comprises the enzymes glutamate oxaloacetate transaminase (GOT) and malate dehydrogenase (MDH), which exist as both cytosolic and mitochondrial isoenzymes, as well as a cytosolic precursor of the mitochondrial MDH isoenzyme [13,14,18].

In proliferating cells, and especially in tumor cells, the degradation of the amino acid glutamine, known as glutaminolysis, is another important metabolic pathway for regenerating energy and cell building blocks, in addition to glycolysis. Correspondingly, high glutaminolysis rates have been described for PC-3 and PC-3M prostate cancer cells [19,20]. In glutaminolysis, glutamine is de-aminated to glutamate. Glutamate is then converted to α-ketoglutarate via three possible reactions (glutamate dehydrogenase, glutamate oxaloacetate transaminase and glutamate pyruvate transaminase) and enters the citric acid cycle. Glutamine itself and the amino acids glutamate and aspartate, which are synthesized in glutaminolysis, are essential precursors for de novo nucleic acid synthesis, glutathione synthesis or serine synthesis. While glycolytic energy production takes place in the cytosol and is independent of oxygen supply, enzymes of glutaminolysis are mainly localized within the mitochondria and the energy regeneration is coupled with mitochondrial respiration [21,22].

Due to the mediating role of mtG3PDH between cytosolic and mitochondrial pathways, we investigated whether the inhibition of mtG3PDH using two commercially available inhibitors affects the conversion rates of glycolysis and glutaminolysis and thus, prostate cancer cell proliferation. We first investigated RH02211, which has been described to inhibit PC-3 cell proliferation at low µM concentrations in a pyruvate-containing medium [23]. We confirmed the inhibition of cell proliferation by RH02211 and investigated the impact of RH02211 on glycolytic and glutaminolytic conversion rates, selected isoenzyme equipments, oxygen consumption and ROS production in PC-3 prostate carcinoma cells. PC-3 cells were chosen due to their high mtG3PDH activity [1]. The reduction of pyruvate to lactate via the LDH reaction represents an alternative metabolic pathway for recycling cytosolic NADH + H^+^ to NAD^+^ through the glycerol 3-P shuttle. Indeed, in addition to the first description from Singh, 2014 we could show that high pyruvate concentrations in the medium weakened the RH0211-induced inhibition of PC-3 cell proliferation [23]. Furthermore, our experiments revealed that extracellular pyruvate abolished the RH02211-induced increase in glycolysis observed in PC-3 cells cultivated in pyruvate-low medium. Notably, culturing PC-3 cells in the presence of 21% or 1.5% oxygen had no effect on the inhibitory action of RH02211 on cell division.

To corroborate our findings, we examined a second mtG3PDH inhibitor (iGP-1) [24] on two selected key targets of RH02211 in PC-3 cells: namely (i) the inhibition of cell proliferation depending upon the pyruvate concentration in the medium and (ii) the effect on glycolytic and glutaminolytic conversion rates under physiological pyruvate concentrations. Finally, inhibition experiments with RH02211 and iGP-1 using DU145 prostate cancer cells revealed that the inhibition of cell proliferation observed in PC-3 cells also occurred in an attenuated form in prostate carcinoma cells with lower mtG3PDH activity [1]. The lack of pyruvate influence on the inhibition of cell proliferation by RH02211 and iGP-1 in DU145 cells correlated with higher H-type LDH and a lower amount of the mitochondrial oxaloacetate transaminase isoenzyme in the DU145 cell line compared to PC-3 cells.

## Materials and methods

### Cell culture

The prostate cancer cell lines PC-3 and DU145 were obtained from CLS Cell Line Service GmbH, Eppelheim, Germany. Both cell lines were cultured in pyruvate-free DMEM-A14430 medium supplemented with 15 mM glucose, 2 mM glutamine, 10% (v/v) FBS superior from Biochrom, Berlin, Germany, 1% (v/v) streptomycin and penicillin (Gibco, Life Technologies, Paisley, UK) and 0.022 mM phenol red (Sigma-Aldrich, München, Germany). In the pyruvate-free medium the pyruvate concentration was 0.015 mM, which originated from the supplemented FBS. The cells that were cultured in medium with 0.015 mM pyruvate are called pyruvate-low cells in this work. In the experiments with pyruvate supplementation (pyruvate-high cells), the final pyruvate concentration in the medium was 2.0 mM. The cells were cultured in presence of 5% CO_2_ and 21% or 1.5% O_2_, respectively. For the low oxygen concentrations cells were cultivated and passaged in an *In VivO*_*2*_ *chamber* from Baker Ruskinn, Sanford, Maine, USA. For the proliferation and flux experiments, 24-well plates were used, and experiments were started with 20,000 cells/well (PC-3 cells) and 40,000 cells/well (DU145 cells). For mass spectrometry measurements of intracellular metabolite concentrations 200,000 cells were seeded in 6-well plates. For the gel permeation, isoelectric focusing and high-resolution respirometry experiments cells were cultured on 15-cm diameter dishes, and experiments were started with 1.5 million cells/dish in the case of PC-3 cells and 2 million cells/dish in the case of DU145 cells.

In the experiments with 1.5% oxygen and/or 2.0 mM pyruvate, the cells were initially pre-cultivated for three days under the respective cultivation conditions without supplementation of inhibitors or vehicles in the medium to adapt the cells to these conditions. After the subsequent passaging of the cells, in the case of the cell proliferation and metabolic flux experiments the inhibitors or vehicle (DMSO) were added immediately into the medium and the cells were cultivated for another 96 hours under the chosen conditions. In the case of intracellular measurements, the inhibitors or vehicle were supplemented 24 hours after cell passage, and cells were harvested after a further 24 hours in the mass spectrometry experiments and after 48 hours in the isoelectric focusing and gel permeation experiments. Cells were counted in presence of Trypan Blue (Sigma-Aldrich Chemie, München, Germany) by a cell counter (Countess II FL – Thermo Fisher Scientific – Life Technologies, Waltham, USA). iGP-1 was purchased from Merck, Sigma-Aldrich Chemie, München, Germany (order no.: 530655); RH02211 from Thermo Fisher Scientific, Schwerte, Germany (order no.: RH02211SC). Both inhibitors were dissolved in DMSO. The DMSO (vehicle) concentration of the controls (= mock-treated controls) corresponded to the DMSO concentration in the cell cultures with the highest concentration of the respective inhibitor in the respective experiments (300 µM iGP-1: 0.19% DMSO; 400 µM iGP-1: 0.25% DMSO; 7 µM RH02211: 0.0035% DMSO and 16 µM RH02211: 0.008% DMSO).

### Metabolic flux in cell culture supernatants

Cells were cultivated in 24-well plates. Every 24 hours, cell culture supernatants from individual wells were stored at −80 °C, and the number of cells in the corresponding well was counted in the presence of trypan blue. The frozen medium supernatants were heated for 15 min at 95°C and subsequently centrifuged at 8000 *g* for 5 min. Glucose, lactate, pyruvate, alanine, serine, glutamine, and glutamate concentrations were determined photometrically using a Respons® 920 bench top analyzer from Diasys Deutschland, Flacht, Germany, as previously described [25,26]. The conversion rates of the metabolites were calculated in [nmol/(h•10^4^ cells].

### Mass spectrometry to determine intracellular metabolite concentrations

Cell plates were placed on dry ice, and 200 µL of metabolite extraction buffer (MEB)/10^6^ cells was added to each well. After 15 minutes of incubation on dry ice, the cells were scraped. Intracellular fractions were incubated in a thermomixer at max speed for 15 minutes at 4 °C and then incubated for 1 hour at −20°C. Proteins were then pelleted by centrifuging samples at 13,000 rpm for 10 minutes at 4°C and supernatants were transferred into glass vials and stored at −80°C until further analysis. LC-MS chromatographic separation of metabolites was achieved using a Millipore SequantZIC-pHILIC analytical column (5 µm, 2.1 × 150 mm) equipped with a 2.1 × 20 mm guard column (both with 5 µm particle size) and a binary solvent system. Solvent A was 20 mM ammonium carbonate, 0.05% ammonium hydroxide; Solvent B was acetonitrile. The column oven and autosampler tray were held at 40°C and 4 °C, respectively. The chromatographic gradient was run at a flow rate of 0.200 mL/min as follows: 0–2 min: 80% B; 2−17 min: linear gradient from 80% B to 20% B; 17–17.1 min: linear gradient from 20% B to 80% B; 17.1–22.5 min: hold at 80% B. Samples were randomized and analyzed with LC–MS in a blinded manner with an injection volume of 5 µL. Pooled samples were generated from an equal mixture of all individual samples and analyzed at regular intervals interspersed throughout the sample sequence as a quality control. Metabolites were analyzed using a Thermo Scientific Q Exactive Hybrid Quadrupole-Orbitrap Mass Spectrometer (HRMS) coupled to a Dionex Ultimate 3000 UHPLC. The mass spectrometer was operated in full-scan, polarity-switching mode, with the spray voltage set to +4.5 kV/-3.5 kV, the heated capillary held at 320 °C, and the auxiliary gas heater held at 280 °C. The sheath gas flow was set to 25 units, the auxiliary gas flow was set to 15 units, and the sweep gas flow was set to 0 unit. HRMS data acquisition was performed in a range of *m/z* = 70–900, with the resolution set at 70,000, the AGC target at 1 × 10^6^, and the maximum injection time (Max IT) at 120 ms. Metabolite identities were confirmed using two parameters: (1) precursor ion m/z was matched within 5 ppm of theoretical mass predicted by the chemical formula; (2) the retention time of metabolites was within 5% of the retention time of a purified standard run with the same chromatographic method. Chromatogram review and peak area integration were performed using the Thermo Fisher software Tracefinder 5.0 and the peak area for each detected metabolite was normalized against the total ion count (TIC) of that sample to correct any variations introduced from sample handling through instrument analysis. The normalized areas were used as variables for further statistical data analysis.

### HPLC to determine the energy charge and purine: Pyrimidine ratio

Cells were washed three times with ice-cold PBS and immediately homogenized in ice-cold 0.6 N HClO_4_. After centrifugation (20 minutes, 40,000 *g*, 4°C), supernatants were adjusted with ice-cold 0.6 N KOH to pH 5–6. To remove KClO_4_, a second centrifugation was performed. The supernatants were lyophilized (0.3 mbar, −5°C, 20 hours; Alpha 1–2 LD plus freeze drying apparatus, Martin Christ, Osterode, Germany) and stored at −80°C until the measurements. Unless otherwise noted, all operations were carried out on ice. Nucleotides were measured by reverse-phase ion-pair liquid-chromatography using an EC 100/3 Nucleodur C18 gravity column (Macherey-Nagel, Düren, Germany). In the mobile phase, a buffer gradient of two base solutions (buffer A: 50 mM KH_2_PO_4_ and 2.5 mM tetra-butylammonium hydrogen sulfate [TBA-HS]; buffer B: 70 mM KH_2_PO_4_, 3.5 mM TBA-HS, and 30% v/v methanol) was used to separate nucleotides. Quantification of the nucleotides was carried out by integration of the peak areas of sample and standard nucleotides using Clarity Chromatography software (Data Apex,Praque, Czech Republic). Nucleotide concentrations were normalized to the protein content of the cell pellets from the first centrifugation step, as measured with the Pierce BCA Protein Assay (Thermo Scientific, Rockford, IL, USA).

### Isoelectric focusing to determine the isoenzyme equipment of LDH, GOT and MDH as well as the isoelectric points of key metabolic enzymes

Cells were extracted in a homogenization buffer containing 10 mM Tris, 1 mM NaF, 1mM EDTA-Na_4_ and 1 mM mercaptoethanol, pH 7.4. Isoelectric focusing was performed using a linear gradient of glycerol (50% to 0% (v/v)) and ampholines (pI 3.5–10.5). The column was run at 400 V for the first hour and 1,200 V for the remainder of 23 h with 4°C cooling water. The column was emptied at a constant rate via a peristaltic pump. In the fractions (1.5 mL volume) pH values and enzyme activities were measured using the Respons® 920 bench top analyzer from DiaSys Deutschland, Flacht, Germany at a wavelength of 340 nm and 37°C as previously described [14,25,27].

### Gel permeation to determine the tetramer : dimer ratio of pyruvate kinase M2

Cells were extracted in a homogenization buffer containing 10% (v/v) glycerin, 0.1 M K_2_HPO_4_/ KH_2_PO_4_ buffer, 50 mM NaC1, l mM 1.4-dithiotreit, 1 mM mercaptoethanol, 1 mM ε-aminocaproic acid, and 0.2 mM phenylmethylsulfonylfluoride. The homogenates were passed over a gel permeation column (HiLoadTM26/60 SuperdexTM 200 (prep grade) from Pharmacia/LKB). In the fractions (1.5 mL) pyruvate kinase activities as well as the activities of the molecular weight markers LDH and MDH were measured using a Respons 920 bench top analyzer from DiaSys Deutschland, Flacht, Germany at a wavelength of 340 nm and 37°C.

### High-resolution respirometry to determine oxygen consumption and H_2_O_2_ production

Cells were cultivated for 96 hours in the presence of RH02211 (16 µM) or the corresponding DMSO concentration. Thereafter, PC-3 cells were trypsinized, counted in the presence of Trypan blue, and resuspended in PBS. Respiratory measurements were performed with samples exhibiting 90% or higher cell viability. To measure O_2_ consumption and H_2_O_2_ production 0.7 ⋅ 10^6^ cells/mL were added into the O2k chambers (Oroboros Instruments, Innsbruck, Austria) which contained 2 mL mitochondrial respiration medium (MiR05-Kit from Oroboros Instruments, Innsbruck, Austria: 110 mM D-sucrose, 60 mM lactobionic acid, 20 mM Hepes, 20 mM taurine, 10 mM KH_2_PO_4_, 3 mM MgCl_2_, 500 µM EGTA and 1 g/L BSA pH adjusted with KOH to pH 7.1). 15 µM diethylenetriaminepentaacetic acid (DTPA), 5 U/mL superoxide dismutase, and 1 U/mL horseradish peroxidase were added to combine measurements of respiration and H_2_O_2_ flux in the FluoRespirometer [28]. During the measurements, the oxygen concentration within the chambers was maintained between 30 and 60 µM, which is closer to tissue levels [29]. The following chemicals were titrated sequentially to measure oxygen consumption and H_2_O_2_ production in different respiratory states, with final concentrations: 10 µM AmplexUltraRed, 0.5 µM rotenone, 10 mM glycerophosphate or 10 mM succinate, respectively, 2.5 mM adenosine diphosphate (ADP), 10 µg/mL digitonin, 15 nM oligomycin, 1–3 µM carbonyl cyanide 3-chlorophenylhydrazone (CCCP) and 2.5 µM antimycin A. The corresponding inhibitor/vehicle concentrations used during the 96-hour incubation were also titrated into the O2k chambers at the beginning of each measurement (ROUTINE + inhibitor) to maintain the same conditions as during cell culture. At the end of the measurements, 30 µM RH02211 was added into the experimental chambers of the control and treated cell samples in order to test the acute effect of RH02211 on both conditions. Data acquisition and analysis was carried out with the software DatLab 7.4 (Oroboros Instruments, Innsbruck, Austria), specifically developed for high-resolution respirometry with the O2k-FluoRespirometer.

### Statistical analysis

#### IC_50_ – IC_90_ values.

A logistic equation model via nonlinear regression method was fitted to the proliferation data to calculate the IC_50_ and IC_90_ values of RH02211 in pyruvate-high PC-3 cells. To calculate the IC_50_ and IC_90_ values of RH02211 in pyruvate-low PC-3 cells, a logistic equation model incorporating a baseline was fitted to the proliferation data using the square root transformation of the drug doses as independent variable (BMDPAR program). For both analyses, the resulting equation for the desired cell count reduction was solved. An estimate and an asymptotic standard error of the parameter of interest were determined by the maximum likelihood method. The statistical comparison of the estimated values between drugs or normoxia vs. hypoxia was then carried out using the asymptotic U test.

#### Metabolic flux in cell culture supernatants.

In a first step, one-way analysis of covariance (ANCOVA) was used to check whether the metabolite conversion rates of the individual test groups were dependent on the cell density in the wells (S1 Fig). If there was a cell density dependence, the metabolic conversion rates of the respective test groups were adjusted to a global mean cell density. In the case of linear relationships, for the statistical analysis the original values were used. In the case of exponential dependencies, the individual values were transformed using logarithms. In the latter cases, the reported means ±SEM correspond to the delogarithmic form of the originally logarithmic values. ANCOVA was used to test both the homogeneity of the slopes and the cell density dependencies among the test groups as well as the differences between the adjusted means. In cases where there was no statistical difference between the slopes, the result of the test for differences between adjusted means from the ANCOVA was used. In those cases where the slopes were significantly different, adjustment to the same mean cell density was done by applying individual slopes. To compare two groups, t-tests were performed using the manually adjusted values (resulting from different slopes). A one-way ANOVA (analysis of variance) + Tukey’s test was performed to compare three groups.

#### Intracellular metabolites by mass spectrometry.

Since the data did not show a normal distribution the Wilcoxon Test using the statistical software package SAS 9.4 [30] was used. The data are shown as median with minimal and maximal values. The significance level was set to p < 0.05.

#### HPLC.

The statistical analysis for evaluating the impact of extracellular pyruvate and the mitochondrial glycerol-3-phosphate inhibitor RH02211 on nucleotides was performed using the Wilcoxon test with the statistical software package SAS 9.4 [30]. The significance level was set to p < 0.05.

#### Pyruvate effect on cell proliferation, enzyme activities and IEPs.

The statistical analysis for evaluating the impact of extracellular pyruvate on PC-3 cell proliferation in the presence of 21% O_2_ and 1.5% O_2_ was performed using the Student’s t-test and the Mann-Whitney test in GraphPad Prism 9 (San Diego, CA, USA). Unpaired Student’s t-test was performed by using GraphPad Prism 9 to evaluate the significancy of total enzymes activities as well as of the composition of the isoenzyme equipment (AUC). The data are shown as mean values with SEM. The significance level was set to p < 0.05.

#### O_2_ consumption and H_2_O_2_ production.

The results correspond to the median values with interquartile range. The Student’s t-test with Mann-Whitney test was used for non-parametrical data analysis.

## Results

### Inhibition of PC-3 cell proliferation by RH02211 depending upon the pyruvate concentration in the medium

RH02211 induced a dose-dependent inhibition of PC-3 cell proliferation (S2A and S2B Figs). Notably, increasing pyruvate levels in the medium weakened the inhibitory effect of RH02211, as evidenced by a significant increase in the IC_50_ and IC_90_ values of RH02211 compared to low pyruvate conditions (Table 1A). Since mtG3PDH is part of the mitochondrial respiration chain, we also investigated the impact of RH02211 on cell proliferation depending on the oxygen supply (21% oxygen versus 1.5% oxygen). Oxygen levels did not influence the effect of RH02211 on PC-3 cell proliferation (Table 1A). Therefore, all following experiments with RH02211 were performed in the presence of 21% oxygen. We used 7 µM RH02211, corresponding to the mean IC_50_ value of RH02211 when cultured with 21% or 1.5% oxygen in pyruvate-low medium, and 16 µM, corresponding to 80% inhibition of cell proliferation in pyruvate-low medium in the presence of 21% oxygen. The inhibitory effect of RH02211 on pyruvate-low PC-3 cell proliferation was reversible when the RH02211-containing medium was replaced with a medium without RH02211 (S3 Fig).

**Table 1:** IC_50_ and IC_90_ values of RH02211 in PC-3 cells (A) and DU145 cells (B). UL = Upper Limits, LL = Lower Limits, n = number of samples. Statistical analysis was performed with the BMDPAR program. Details are described in Materials and Methods. *: p = 0.02. Significances (p values) between PC-3 and DU145 cells: IC_50_ (0.015 mM Pyr) < 0.001, IC_50_ (2.0 mM Pyr) < 0.001, IC_90_ (0.015 mM) = 0.036, IC_90_ (2.0 mM Pyr) < 0.001.

A	PC-3 cells	*IC*_*50*_ (*µM)*						*IC*_*90*_ (*µM)*					
		**21% O**_**2**_			**1.5% O**_**2**_			**21% O**_**2**_			**1.5% O**_**2**_		
		**IC**_**50**_ **value**	UL	LL	**IC**_**50**_ **value**	UL	LL	**IC**_**90**_ **value**	UL	LL	**IC**_**90**_ **value**	UL	LL
	0.015 mM Pyr	**6.1** (n = 63)	7.5	4.9	7.9 (n = 76)	8.9	7.0	17.7 (n = 63)	24.3	12.1	32.4 (n = 76)	37.1	28.0
		|*											
	2.0 mM Pyr	**9.4** (n = 48)	10.5	8.3	9.3 (n = 54)	10.4	8.1	20.6 (n = 48)	23.2	18.0	26.5 (n = 54)	30.5	22.6
B	**DU145 cells**	***IC***_***50***_ **(***µ****M)***						***IC***_***90***_ **(***µ****M)***					
		**21% O**_**2**_						**21% O**_**2**_					
		**IC**_**50**_ **value**		UL	LL			**IC**_**90**_ **value**		UL	LL		
	0.015 mM Pyr	21.4 (n = 54)		25.0	17.9			32.7 (n = 32)		39.8	25.6		
	2.0 mM Pyr	16.9 (n = 48)		20.3	13.5			38.9 (n = 32)		48.0	29.8		

### Impact of RH02211 on PC-3 cell metabolism depending upon the pyruvate concentration in the medium

**Fig 1:**
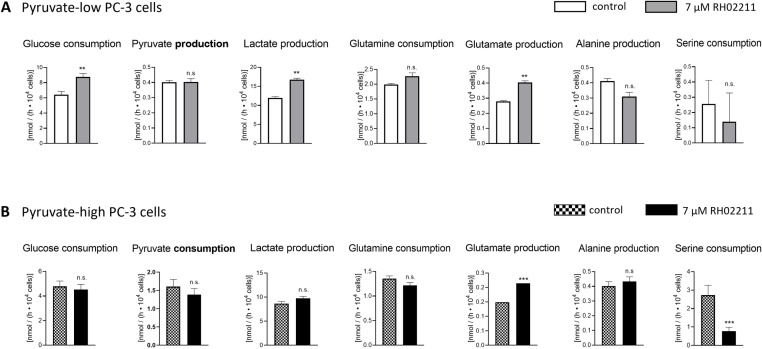
Impact of RH02211 on glycolytic and glutaminolytic conversion rates in cell culture supernatants of PC-3 cells, depending on the pyruvate concentration in the medium. A) pyruvate-low PC-3 cells and B) pyruvate-high PC-3 cells. RH02211 concentration: 7 µM, which corresponds to the IC_50_ value for inhibiting cell proliferation by RH02211. One-way analysis of covariance (ANCOVA) was used to test the homogeneity of slopes and cell density dependencies among the test groups, as well as the differences between the adjusted means. $\overset{―}{x}$ ± SEM. **: p ≤ 0.01 and ***: p ≤ 0.001 versus control cells. Number of values per group ≥ 17.

**Fig 2:**
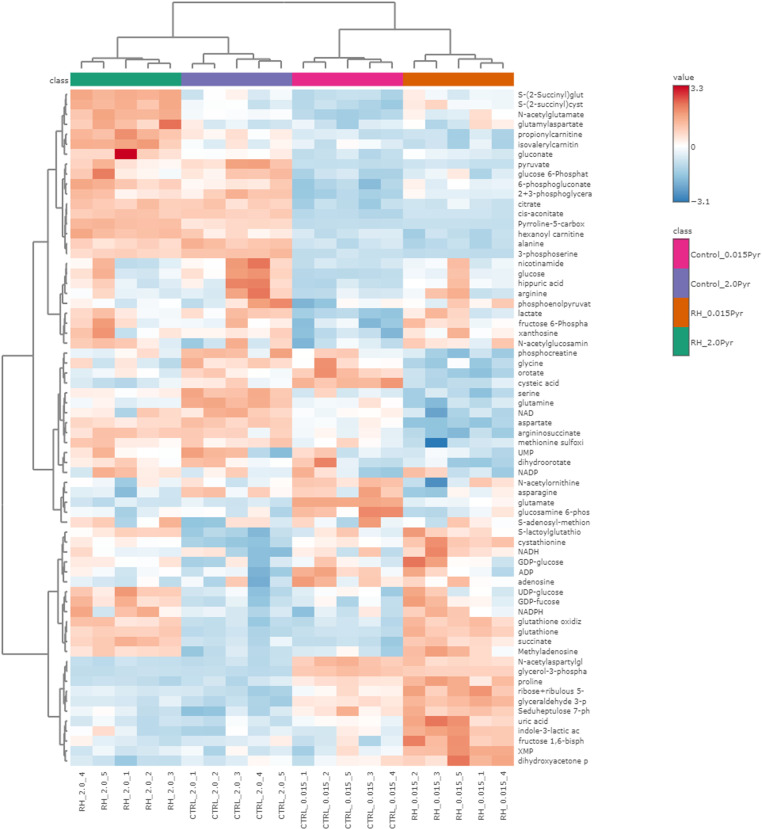
Heatmap showing the impact of RH02211 on intracellular concentrations of metabolic intermediates in PC-3 cells depending upon the pyruvate concentration in the medium. RH02211 concentration: 7 µM.

**Fig 3:**
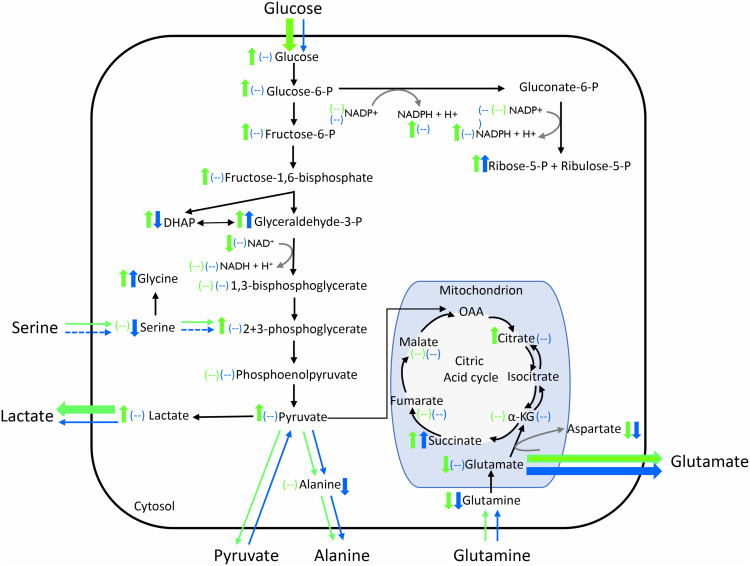
Impact of RH02211 on glycolytic and glutaminolytic conversion rates as well as intracellular concentrations of metabolic intermediates in PC-3 cells depending upon the pyruvate concentration in the medium. RH02211 concentration: 7 µM. Green arrows = impact of 7 µM RH02211 in pyruvate-low cells; blue arrows = impact of 7 µM RH02211 in pyruvate-high cells. Extracellular metabolites: bold arrows = increase of the conversion rate in the presence of 7 µM RH02211 compared to controls; dashed arrows = decrease of the conversion rate in the presence of 7 µM RH02211 compared to control. Intracellular metabolites: ↑ = increase in intracellular concentration; ↓ = decrease in intracellular concentration; (--) = concentrations unchanged.

RH02211 induced a dose-dependent increase in glucose consumption, lactate production, glutamine consumption, glutamate production, and alanine production when cultivated in pyruvate-low medium using inhibitor concentrations of 7 µM and 16 µM (S4 Fig). We next investigated the influence of RH02211 on PC-3 metabolism as a function of the pyruvate concentration in the medium. The RH02211 concentration was 7 µM in pyruvate-low and pyruvate-high cells. Measurements of the metabolic conversion rates in the cell culture supernatants revealed a significant increase in glucose consumption and lactate production in RH02211-treated pyruvate-low PC-3 cells (Fig 1A). In contrast, in pyruvate-high PC-3 cells, the inhibitor had no impact on the glycolytic conversion rates (Fig 1B). Accordingly, in RH02211-treated pyruvate-low cells, but not in their pyruvate-high counterparts, intracellular glucose, glucose 6-P, fructose 6-P, fructose 1,6-P2, the sum of glycerate 2-P and glycerate 3-P, pyruvate, and lactate concentrations increased (Figs 2 and 3, S1 Table). Only glyceraldehyde-3-P concentrations increased in both pyruvate-low and pyruvate-high RH02211-treated PC-3 cells (Figs 2 and 3, S1 Table). The increase of intracellular pentose phosphates suggests that a part of the glucose consumed in pyruvate-low and pyruvate-high RH02211-treated PC-3 cells was likely channeled into the pentose phosphate pathway (Figs 2 and 3, S1 Table). Pyruvate conversion rates (consumption in pyruvate-high PC-3 cells and production in pyruvate-low PC-3 cells) were not changed by RH02211 (Figs 1 and 3). Interestingly, intracellular pyruvate concentrations increased significantly in RH02211-treated PC-3 cells cultivated in a pyruvate-low medium, but not in a pyruvate-high medium. The significant decrease of serine consumption in pyruvate-high RH02211-treated PC-3 cells correlated with a decrease of intracellular serine concentrations (Figs 1-3, S1 Table).

A common effect of RH02211 in pyruvate-low and pyruvate-high PC-3 cells was an increase in glutamate release, parallel to unchanged glutamine conversion rates, and a decrease in intracellular glutamine and aspartate concentrations, suggesting an impairment of glutaminolysis by RH02211 at both low and high pyruvate cultivation conditions. The intermediates of the citric acid cycle were unaffected or slightly increased in both pyruvate-low and pyruvate-high RH02211-treated cells (Figs 2 and 3, S1 Table). Together with the unchanged energy charge, the intracellular metabolite concentrations do not point to an impairment of the citric acid cycle when RH02211 was supplemented in a concentration of 7 µM independent of the pyruvate supplementation (Figs 2 and 3, S1 and S2 Tables).

### Impact of RH02211 on O_2_ consumption in pyruvate-low and pyruvate-high PC-3 cells

**Fig 4:**
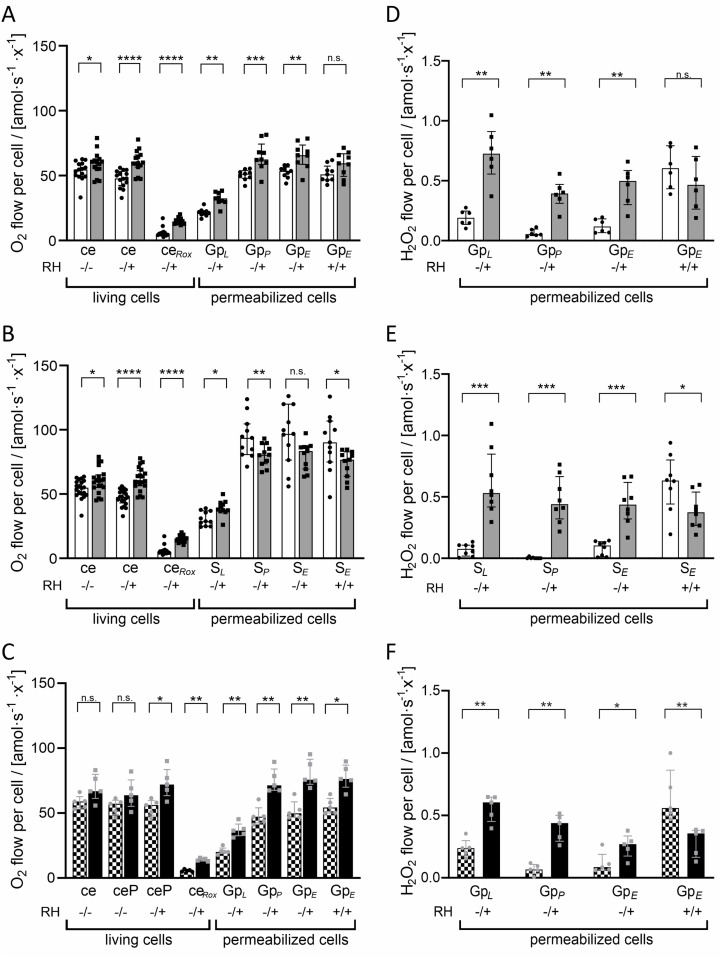
Impact of RH02211 on O_2_ consumption and H_2_O_2_ production in PC-3 cells. **A**, **B**, **D**, **E**: Pyruvate-low PC-3 cells: white bars = control, gray bars = cultivated for 96 hours in the presence of 16 µM RH02211. **C**, **F**: pyruvate-high PC-3 cells: checkered bars = control, black bars = 16 µM RH02211. **A** and **D:** Fuel substrate: 10 mM glycerol 3-P. **B** and **E:** Fuel substrate: 10 mM succinate. **C** and **F**: Fuel substrate: 2 mM pyruvate and 10 mM glycerol-3-P. At the beginning of the measurements (ROUTINE + RH02211) RH02211 or DMSO were titrated into the O2k chambers in order to maintain the same conditions as during cell culture (compare Materials and Methods). Presence of 16 µM RH02211 represented in the abscissa by “+”. ce = ROUTINE, ceP = acute pyruvate titration in ROUTINE state, ce_*Rox*_ = residual oxygen consumption (after rotenone), Gp_*L*_ or S_*L*_ = LEAK state with glycerol-3-P or succinate, respectively, Gp_*P*_ or S_*P*_ = OXPHOS, Gp_*E*_ or S_*E*_ = ETcapacity. A “+” sign indicates titration of 30 µM RH02211. The Student’s t-test with Mann-Whitney test was used for non-parametrical data analysis; data are shown as median values with interquartile range. *: p < 0.05, **: p ≤ 0.01, ***: p ≤ 0.001, ****: p ≤ 0.0001. n ≥ 6 for pyruvate-low cells in presence of glycerol-3-P; n ≥ 8 for pyruvate-low cells in presence of succinate; n = 5 for pyruvate-high cells in presence of glycerol 3-P.

We then examined the influence of RH02211 on the oxygen consumption rates of the PC-3 cells. Measurement of ROUTINE respiration without the addition of glycerol-3-P or the complex II substrate succinate revealed a significant increase in O_2_ consumption in living pyruvate-low PC-3 cells cultivated for 96 hours in the presence of 16 µM RH02211 (Figs 4A and 4B: ce). Acute titration of RH02211 into the chambers increased the significance of the effect (Figs 4A and 4B: ce -/- to -/+). In contrast, in living PC-3 cells cultivated with 2.0 mM pyruvate, O_2_ consumption in ROUTINE respiration was not impaired by 96 hours treatment with 16 µM RH02211 neither before (Fig 4C: ce) nor after acute titration of pyruvate (Fig 4C: ceP), which is consistent with the minor impact of RH02211 on the conversion rates in the culture medium and the intracellular metabolite concentrations in pyruvate-high PC-3 cells (Figs 1–3). In both pyruvate-low and pyruvate-high RH02211-treated PC-3 cells, O_2_ consumption increased after permeabilization of the cells when glycerol-3-P was used as fuel substrate (Figs 4A and 4C: Gp_*P*_). To our surprise, when succinate was used as a fuel substrate of the mitochondrial respiratory system, a decrease of O_2_ consumption was induced by RH02211 in permeabilized pyruvate-low cells (Fig 4B: S_*P*_).

### Impact of RH02211 on H_2_O_2_ production in pyruvate-low and pyruvate-high PC-3 cells

In permeabilized pyruvate-low PC-3 cells in the presence of either glycerol-3-P or succinate as fuel substrate and in permeabilized pyruvate-high PC-3 cells in the presence of glycerol-3-P as fuel substrate RH02211 induced an increase of H_2_O_2_ production (Figs 4D–4F: Gp_*L*_, Gp_*P*_, Gp_*E*_, S_*L*_, S_*P*_, S_*E*_; + /-). Since the measurements were performed in permeabilized cells to which RH02211 was acutely titrated during ROUTINE respiration, it was not possible to distinguish whether the increase in H_2_O_2_ production was induced by the 96-hour treatment or by the acute titration of the inhibitor. However, the acute titration of RH02211 to the control cells induced an increase in H_2_O_2_ production in pyruvate-high PC-3 cells when glycerol-3-P was used as a fuel substrate, and in pyruvate-low cells when succinate was used as a fuel substrate (Figs 4E and 4F: Gp_*E*_ and S_*E*_; + /+). A control background experiment without cells confirmed that the increase in H_2_O_2_ production was not due to a reaction between RH02211 and other chemicals used in the experiments (S5 Fig).

### Impact of RH02211 on mtG3PDH

**Fig 5:**
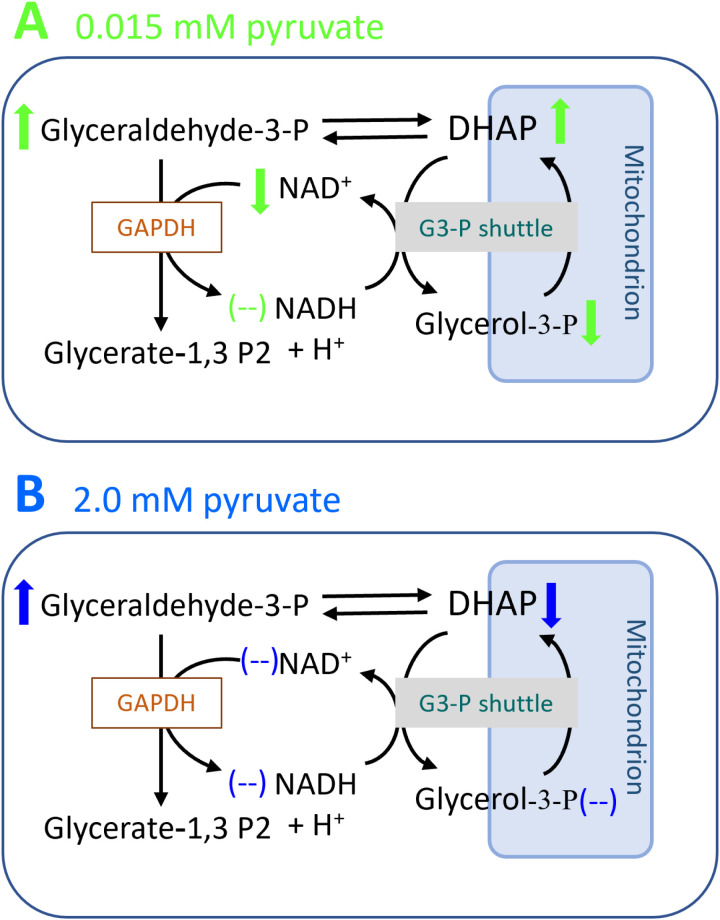
Metabolic scheme illustrating the impact of RH02211 on the intracellular concentrations of metabolites involved in the glycerol-3-phosphate shuttle. **A:** in pyruvate-low and **B:** in pyruvate-high PC-3 cells. RH02211 concentration: 7 µM. Original values on S1 Table.

We measured the intracellular concentrations of glycerol-3-P and DHAP and found significant changes in RH02211-treated PC-3 cells, which indeed indicate an impact of RH02211 on the glycerol-3-P shuttle. In pyruvate-low RH02211-treated PC-3 cells the concentration of glycerol-3-P, the substrate of mtG3PDH, decreased and the concentration of DHAP, the product of the mtG3PDH reaction, increased which rather suggests an activation of mtG3PDH in our experimental design with long term exposition of the cells with the drug (Figs 2 and 5, S1 Table). On the other hand, the increase in DHAP and decrease in glycerol-3-P, together with the decrease in NAD^+^ concentrations, point to an impairment of cG3PDH by RH02211. In contrast to what was observed in pyruvate-low conditions, the significant reduction in DHAP in the pyruvate-high RH02211-treated PC-3 cells is consistent with an inhibition of mtG3PDH. Interestingly, in pyruvate-low PC-3 cells, RH02211 treatment was associated with an increase of both DHAP and glyceraldehyde-3-P concentrations, while in pyruvate-high cells in the presence of RH02211, glyceraldehyde-3-P was the only glycolytic intermediate whose concentration increased, and DHAP was the only glycolytic intermediate whose concentration decreased (Fig 5 and S1 Table). Pyruvate supplementation without RH02211 induced a decrease of glyceraldehyde-3-P without affecting the DHAP concentration (S8 Fig and S3 Table).

### Impact of the mtG3PDH inhibitor iGP-1 on cell proliferation and metabolism of PC-3 cells

**Fig 6:**
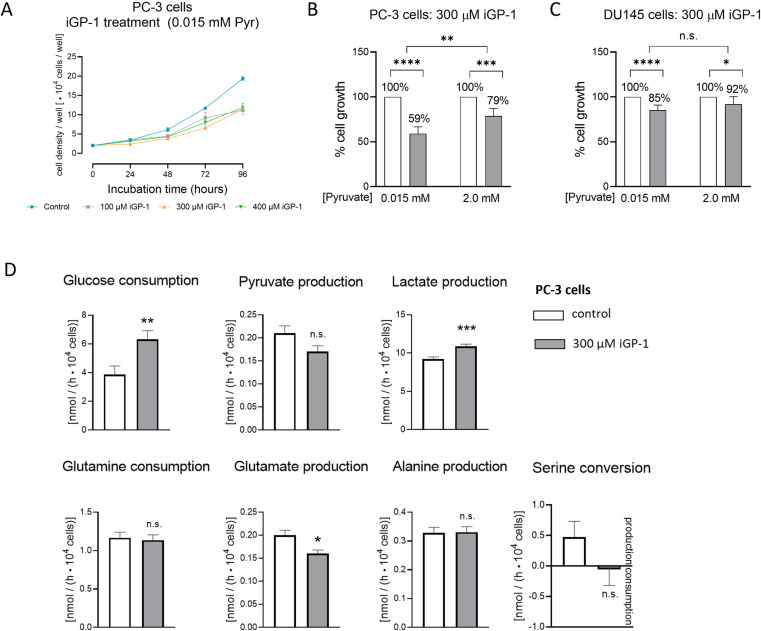
Impact of iGP-1 on cell proliferation of PC-3 and DU145 cells and on the metabolism of PC-3 cells. **A:** Dose-finding study and time course of iGP-1-induced PC-3 inhibition in a pyruvate-low medium. **B:** Impact of 300 µM iGP-1 on cell proliferation of PC-3 cells depending upon the pyruvate concentration in the medium. **C:** Impact of 300 µM iGP-1 on cell proliferation of DU145 cells depending upon the pyruvate concentration in the medium. **B** and **C:** The mean cell density/well of mock-treated control cells was set to 100%. **D:** Glycolytic and glutaminolytic conversion rates measured in the culture media supernatants of pyruvate-low PC-3 cells. Student’s t-test with Mann-Whitney test was performed to assess significance in B and **C.** Statistical analysis of glycolytic and glutaminolytic conversion rates (D) was performed using ANCOVA. Mean ± SEM, n = 6 (A-B), n = 18 (C). *: p ≤ 0.05, **: p ≤ 0.01, ***: p ≤ 0.001, ****: p ≤ 0.0001.

We then tested a second mtG3PDH inhibitor, iGP-1. In pyruvate-low PC-3 cells, iGP-1 induced an approximately 40% inhibition of cell proliferation, independent of dosage, when concentrations between 100 µM and 400 µM were tested under 21% O_2_ (Fig 6A). Higher concentrations of iGP-1 could not be tested because the concentration of DMSO required to dissolve the inhibitor in higher concentrations (above 0.3% (v/v)) was toxic to PC-3 cells. Notably, 300 µM iGP-1 induced only 20% inhibition of cell proliferation under high-pyruvate conditions, which is consistent with an escape mechanism of extracellular pyruvate for the RH02211-induced inhibition of cell proliferation in PC-3 cells (Fig 6B). The cultivation of PC-3 cells in pyruvate-low medium in the presence of 300 µM iGP-1 was accompanied by a significant increase in glucose consumption and lactate production and by a significant decrease in glutamate production when compared to mock-treated controls. In contrast, no influence of iGP-1 was detected on the conversion rates of pyruvate, glutamine, alanine, and serine (Fig 6D). Together, these results point to an increase in glycolysis and an impairment of glutaminolysis in PC-3 cells inhibited by iGP-1.

### Impact of RH02211 and iGP-1 on cell proliferation of DU145 cells in comparison to PC-3 cells

We next examined the influence of RH02211 and iGP-1 on the proliferation rate of another prostate cancer cell line, DU145. The metastatic potential of DU145 cells is described as moderate compared to that of PC-3 cells [31]. DU145 and PC-3 cells were characterized by approximately the same level of cG3PDH activity, whereas the mtG3PDH activity in DU145 cells is approximately 40% lower compared to PC-3 cells. The ratio between cG3PDH and mtG3PDH is about 7.2 : 1 in PC-3 cells and 10.5 : 1 in DU145 cells [1]. The lower amount of mtG3PDH in DU145 cells correlates with an approximately 3.5 times higher IC_50_ value for RH02211 compared to PC-3 cells when both cell lines were cultivated under low-pyruvate conditions (Tables 1A and 1B). Likewise, the inhibitory effect of iGP-1 on the proliferation of DU145 cells was significantly weaker than on PC-3 cells. 300 µM iGP-1 resulted in 15% inhibition of cell proliferation in pyruvate-low DU145 cells and 41% inhibition in pyruvate-low PC-3 cells (Figs 6B and 6C). Supplementation of pyruvate into the medium was associated with a weakening of the inhibitory effect of iGP-1 and RH02211 on PC-3 cells but not on DU145 cells, which may be correlated with the different LDH isoenzyme equipment of both cell lines (Fig 6C, Tables 1A and 1B). Pyruvate is the substrate of LDH, which recycles NAD^+^ for the cytosolic GAPDH reaction. In comparison to PC-3 cells, DU145 cells were characterized by lower total LDH activity but twice as high proportion of the H-type LDH isoenzyme (ratio of H-type : Hybrids : M-type in DU145 cells is 2.4 : 2.1 : 1.0). In PC-3 cells the M-type of the LDH isoenzyme was predominant (ratio of H-type : Hybrids : M-type is 1.0 : 2.2 : 2.2) (Table 2, S4 and S5 Tables). In addition to differences in mtG3PDH and LDH, our enzyme characterizations revealed differences in the malate-aspartate shuttle enzyme GOT between the two prostate carcinoma cell lines (Table 2). While in PC-3 cells, the percentage of the mitochondrial GOT isoenzyme was approximately twice that of the cytosolic isoenzyme, the ratio of the cytosolic GOT isoenzyme to the mitochondrial GOT isoenzyme was balanced in DU145 cells. There were no differences between PC-3 and DU145 cells in MDH, the second enzyme involved in the malate-aspartate shuttle (Table 2).

**Table 2:** Isoenzyme equipment of lactate dehydrogenase (LDH), glutamate oxaloacetate transaminase (GOT) and malate dehydrogenase (MDH) of pyruvate-low PC-3 and DU145 cells as measured by free flow isoelectric focusing. The percentual amounts were calculated as area under the curve (AUC) in percent of the respective total enzyme activity loaded onto the focusing column. Cyto = cytosolic isoenzyme, mito = mitochondrial isoenzyme, prec = cytosolic precursor of the mitochondrial isoenzyme. Unpaired Student’s t-test was performed to assess significancy. Mean ± SEM. n = 3. P-values (PC-3 vs DU145): LDH H-type and M-type (****) < 0.0001, GOT cyto and mito (**) = 0.0035. In PC-3 cells neither supplementation of extracellular pyruvate nor RH02211 in concentration of 16 µM had an effect on the isoenzymes of LDH, GOT and MDH.

	AUC (%) $\overset{―}{x}$ ± SEM							
	LDH (****)			GOT (**)		**MDH (n.s.)**		
	H-type	Hybrid	M-type	cyto	mito	cyto	prec	mito
PC-3	19 ± 0.6	41 ± 0.5	41 ± 1.2	34 ± 1.9	66 ± 1.9	49 ± 3.1	21 ± 8.1	30 ± 10.0
DU145	43 ± 2.3	39 ± 1.6	18 ± 1.3	51 ± 3.0	49 ± 3.0	53 ± 4.4	24 ± 7.0	23 ± 9.5

Another interesting difference between the PC-3 and DU145 cells concerned M2-PK. When the cells were cultured in pyruvate-low conditions, the tetramer : dimer ratio of M2-PK was 40% : 60% in both cell lines. While the extracellular pyruvate concentration had no impact on the tetramer : dimer ratio of M2-PK in PC-3 cells. In DU145 cells supplementation of 2 mM pyruvate resulted in a shift of the tetramer : dimer ratio towards the dimeric form (Table 3).

**Table 3:** M2-PK tetramer : dimer ratio in PC-3 and DU145 cells depending upon the pyruvate concentration in the medium. Unpaired Student’s t-test was performed to assess significancy. Mean ± SEM. n = 3. * = significance between pyruvate-high DU145 cells versus pyruvate-high PC-3 cells = 0.0407. Supplementation of 16 µM RH02211 had no effect on the M2-PK tetramer : dimer ratio of PC-3 cells. In both pyruvate-low and pyruvate-high PC-3 cells supplementation of 16 µM RH02211 had no effect on the M2-PK tetramer : dimer ratio.

	PC-3 cells		DU145 cells	
Pyruvate (mM)	0.015	2.0	0.015	2.0
	AUC (%) $\overset{―}{x}$ ± SEM			
% Tetra	40 ± 6.2	44 ± 3.5	40 ± 6.1	29 ± 3.5 ^*^
% Dimer	60 ± 6.2	56 ± 3.5	60 ± 6.1	71 ± 3.5 ^*^

## Discussion

In PC-3 prostate cancer cells cultivated in pyruvate-low medium, the mtG3PDH inhibitor RH02211 induced a dose-dependent inhibition of cell proliferation and a dose-dependent increase in glycolysis (S2 and S4 Figs). This increase of glycolysis appears to contradict the positive effect of high glycolytic conversion rates on cell proliferation, as first described by Otto Warburg [32], and confirmed in multiple systems afterwards. Likewise, the unchanged energy charge and the unchanged (ATP + GTP) : (CTP + UTP) ratio in pyruvate-low RH02211-treated PC-3 cells do not indicate an inhibition of cell proliferation (S2 Table). Low proliferation rates have been shown to correlate with a high ratio of purines to pyrimidines in various cell lines [25,33,34]. High concentrations of pyruvate in the medium weakened the growth-inhibitory effect of RH02211 and abolished the observed increase in glycolysis (Figs 1−3, S2 and S4 Figs, Table 1). An increase of glycolysis and a weakening impact of high pyruvate concentrations in the medium on the inhibition of cell proliferation was also observed for the second mtG3PDH inhibitor, iGP-1 (Figs 6B and 6D). The iGP-1 concentration tested in our study was 300 µM, which corresponded to approximately 40% inhibition of cell proliferation (Figs 6A and 6B). For iGP-1, an increase in glycolysis was also described in HDMB03 and D-458 medulloblastoma cell lines when iGP-1 was applied in a concentration of 1 µM and 100 µM in the presence of 6% and 1% oxygen but not 21% oxygen, which correspond to the oxygen concentration in our iGP-1 experiments [35]. However, concerning cell division in HDMB03 and D-458 cells cultivated in the presence of low oxygen (6% or 1%), treatment with 1 µM and 100 µM iGP-1 for 24 and 72 hours significantly increased cell proliferation. In our experiments, low oxygen concentrations (1.5%) did not influence the inhibitory effect of RH02211 on the cell proliferation rate of PC-3 cells (Table 1A). Therefore, all subsequent investigations in our study, including those of iGP-1, were performed in the presence of 21% oxygen. In Med1-medullablastoma cells cultivated in the absence of pyruvate and in the presence of 21% oxygen, 100 µM iGP-1 induced approximately 25% inhibition of cell proliferation, which was associated with a reduction in GLI1 expression, a target of Hedgehog signaling [36]. iGP-1-induced inhibition of cell proliferation or induction of apoptosis was also reported for HCT116 colon cancer cells (1 mM iGP-1) [37], neutrophils in the presence of 1% oxygen (1 mM iGP-1) [38] and in chromosomal instability (CIN) tumors of Drosophila (1 mM iGP-1) [39]. The reported iGP-1 concentrations necessary to inhibit cell proliferation (up to 1mM) are higher than those of RH02211. Still, our experiments revealed a consistent inhibitory effect of iGP-1 on cell proliferation over a wide concentration range of 100–400 µM. To further develop iGP-1 or its derivatives with regard to therapeutic approaches, monitoring the intracellular iGP-1 concentrations achieved will provide more clarity on dosing. Due to the observed interesting influence of extracellular pyruvate on the inhibitory effect of RH02211 on PC-3 cell proliferation, we focused the further course of our study on investigating pyruvate-dependent metabolic changes under RH02211.

Most commercially available cultivation media for tumor cells contain 1–5 mM pyruvate to enhance cell survival and proliferation. In fact, in PC-3 cells, supplementation of 2 mM pyruvate during two passages induced a significant increase of PC-3 cell proliferation in the presence of 21% as well as 1.5% O_2_ (S6 Fig). A pyruvate-induced increase of cell proliferation is also documented for MCF-7, MB231 and T-47D breast cancer cells [40]. The comparison of pyruvate-low and pyruvate-high PC-3 cells revealed a boosting impact of extracellular pyruvate on cells cell metabolism, which shifted from pyruvate production when cultivated in a pyruvate-low medium to pyruvate consumption when cultivated in a pyruvate-high medium (S7 and S8 Figs, S3 Table). The significant reduction in lactate release in pyruvate-high PC-3 cells indicates that extracellular pyruvate was not predominantly used for the release of cytosolic hydrogen as lactate but was channeled into other metabolic pathways, such as the citric acid cycle. In murine squamous cell carcinoma SCCVII and human colon cancer HT29 cell lines, basal oxygen consumption increased when cultured in media containing 0.2–2.0 mM pyruvate compared to the corresponding cell lines cultivated in the absence of pyruvate, indicating that pyruvate acts as a booster of mitochondrial energy metabolism [40,41]. Accordingly, in pyruvate-high conditions, intracellular levels of pyruvate, lactate, citrate, cis-aconitate, α-ketoglutarate, fumarate, and malate were significantly increased compared to those in pyruvate-low conditions in PC-3 cells (S8 Fig, S3 Table).

Cytosolic pyruvate is transported into the mitochondria via the mitochondrial pyruvate carrier (MPC). The regulation of MPC expression by the androgen receptor (AR) is a crucial control element in the metabolism of androgen receptor-positive and castration-resistant prostate cancer cells, which enables the growth of these tumor cells [42]. Accordingly, inhibition of MCP induced a dose-dependent decrease in the proliferation of hormone-responsive PCa cell lines, including LNCaP, VCaP, and C4-2, as well as the castrate-resistant ABL model, which maintains MPC protein expression during treatment with androgens and anti-androgens, but not in AR-negative prostate cancer cell lines PC-3 and DU145 [42]. The breakdown of extracellular pyruvate via the citric acid cycle and oxygen-dependent oxidative phosphorylation provides 12.5 moles of ATP per mole of pyruvate, while the oxygen-independent cytosolic conversion of extracellular pyruvate to lactate recycles one mol of NAD^+^ per mol pyruvate but does not provide ATP. The larger supply of energy resulting from mitochondrial pyruvate degradation may explain the observed parallel reduction in the conversion rates of glucose and glutamine in pyruvate-high PC-3 cells compared to their pyruvate-low counterparts (S7 and S8 Figs). The stable energy charge in pyruvate-low and pyruvate-high PC-3 cells reflects an energetically balanced metabolic adaptability of the cells independent from the pyruvate supply (S2 Table).

The physiological concentration of pyruvate in the blood is about 0.1 mM [43]. Since tumors are often poorly vascularized compared to healthy tissue, it is assumed that the tumor interstitial fluid (TIF) is poorer in nutrients such as pyruvate and enriched with more metabolic end products than plasma. At the time of writing this manuscript, we did not find any published data dealing with pyruvate concentrations in the microenvironment of prostate cancer in situ. A comprehensive comparative determination of more than 100 metabolites in the plasma and TIF isolated from autochthonous and transplantation models of murine lung and pancreatic ductal adenocarcinomas showed that the metabolite composition of the TIF depends on the localization of the tumor within the tissue, on the diet of the animals, and from the metabolic properties of the tissue from which the tumor originated [44]. Notably, the TIF of pancreatic adenocarcinomas exhibited lower levels of pyruvate compared to plasma, suggesting that this metabolite concentration may be below 0.1 mM within the TIF. Yet, in the interstitial fluid of lungs of healthy BALB/c mice, the pyruvate: glutamine ratio was approximately three times higher compared to the animal´s blood plasma (estimated from Fig 3 in [45]). In the highly metastatic 4T1 breast cancer mouse model of the same study, the intracellular pyruvate levels were increased in the lung metastases compared to their primary breast cancers, which led the authors to conclude that the TME in the lung promotes increased pyruvate carboxylase-dependent anaplerosis due to the higher pyruvate availability in the lung [45]. Based on the physiological pyruvate concentration of about 0.1 mM in plasma, it can be assumed that the pyruvate concentrations in the TIF of individual organs, such as the lung, may be somewhat higher than the plasma levels. Still, it may not reach the high concentrations of 1–5 mM of commercially culture media [46]. Therefore, depending on the individual metabolism of the tumor cells and the affected organ, the pyruvate present in the TME can have different significance for the survival and spread of the tumor cells and thus, also for therapeutic approaches that intervene in the tumor metabolism, such as mtG3PDH inhibition.

The only reductions in metabolic conversion rates and intracellular metabolite concentrations, which could be related to the inhibition of cell proliferation of both pyruvate-low and pyruvate-high conditions in PC-3 cells by RH02211, were observed within glutaminolysis and the intracellular concentrations of the amino acids glutamine, glutamate, aspartate (Figs 1–3, S1 Table). In prostate tumors, ASCT2 was identified as a key glutamine transporter. In AR-positive LNCaP prostate cancer cells, 5α-dihydrotestosterone (DHT) significantly increased the expression levels of ASCT2 and glutaminase (GLS), the enzyme responsible for the deamination of glutaminase to glutamate. In contrast, in AR-negative PC-3 and DU145 cells, DHT had no effect on ASCT2 and GLS expression [47], which indicates a role of AR in the regulation of glutaminolysis in AR-positive prostate cancer cells. Glutamine starvation and inhibition of GLS reduced cell viability of AR-positive as well as AR-negative prostate cancer cells lines, whereby the effect on AR-negative cell lines was more pronounced in comparison to AR-positive prostate cancer cell lines [42,47]. However, in contrast to RH02211, in iGP-1-treated pyruvate-low PC-3 cells, the metabolic conversion rates indicate an activation of glutaminolysis (Fig 6D), which suggests a partly different mode of action of RH02211 and iGP-1 in PC-3 cells. In ABL prostate cancer cells, metabolomic analyses using U^13^C-labelled glutamine 7% of the α-ketoglutarate pool and 20% of the citrate pool originated from extracellular glutamine. Inhibition of MPC increased the proportion of α-ketoglutarate and citrate formed from extracellular glutamine to 37% and 68%, respectively [42]. Together, these results reflect the high complexity and networking of the interactions between the different metabolic pathways and their regulators in tumor cells.

Measurements of mitochondrial respiration rates reflected an increase of oxygen consumption in intact pyruvate-low PC-3 cells cultivated for 96 hours in the presence of RH02211 in the ROUTINE state before and after acute titration of RH02211 (Figs 4A and4B: ce, -/- and -/+). Similar to our results on glycolysis, the RH02211-induced increase in oxygen consumption was prevented in intact PC-3 cells in high-pyruvate medium, both before and after acute pyruvate titration (Fig 4C: ce and ceP). In permeabilized RH02211-treated PC-3 cells, oxygen consumption increased in the presence of glycerol-3-P independent of the pyruvate concentration (Figs 4A and 4C: Gp_*L*_, Gp_*P*_, Gp_*E*_). When succinate, the substrate of complex II, was used as a fuel substrate of the mitochondrial respiratory system, a decrease of O_2_ consumption was induced by RH02211 in permeabilized pyruvate-low cells (Fig 4B: S_*L*_and S_*P*_), suggesting an inhibitory effect of RH02211 on respiration supported by the succinate pathway. Some nonspecific effects on succinate dehydrogenase are also described for the mtG3PDH inhibitor iGP-1 when mitochondria isolated from brown adipose tissue were treated with 0–100 µM iGP-1 in presence of 10 mM succinate [48].

With glycerol 3-P as substrate, H_2_O_2_ production increased in pyruvate-low and pyruvate-high RH02211 treated cells (Figs 4D and 4F: S_*L*_, S_*P*_, S_*E*_; -/+). In contrast to published oxidative stress models [49–53] high extracellular pyruvate concentrations did not mediate protection against the RH02211-induced increase in H_2_O_2_ production (Fig 4F). Since the H_2_O_2_ measurements were performed in permeabilized cells to which RH02211 was acutely titrated during ROUTINE, it is not possible to distinguish whether the increase in H_2_O_2_ production was induced by the 96-hour treatment or by the acute titration of the inhibitor. mtG3PDH has been shown to participate in mitochondrial ROS production in both the mitochondrial matrix and the intermembrane space [1]. For this reason, we expected that RH02211, as an inhibitor of mtG3PDH, should lead to a reduction in H_2_O_2_ concentration in our experiments with PC-3 cells. In fact, in the first description of the inhibitor, a drop in H_2_O_2_ production is described after short-term (15 minutes) application of PC-3 cells with 1–30 µM RH02211 [23]. The experiments in our study differ from the Singh’s study in three aspects. (i) In our study, the PC-3 cells were cultured significantly longer in the presence of the inhibitor (96 hours, compared to 15 minutes in Singh’s 2014 experiments [23]). (ii) Singh measured extracellular H_2_O_2_ using the HRP/AmplexUltraRed assay in the absence of exogenous superoxide dismutase by the EnVision 2102 multilabel reader, whilst we used the HRP/Amplex Red assay at saturating exogenous SOD levels in combination with the O2k-FluoRespirometer (Oroboros Instruments, Innsbruck, Austria). The HRP/Amplex Red assay detects H_2_O_2_ that is formed by spontaneous dismutation of superoxide through endogenous SOD. The addition of exogenous SOD at saturating levels ensures that all reactive oxygen species that may have been produced by the cells are converted to H_2_O_2_. Besides, SOD minimizes the formation of UltroxRed in a photosensitized reaction with NADH and reduces glutathione. (iii) Further, by using the O2k-FluoRespirometer, total cellular H_2_O_2_ production rates were measured since the measurements were performed after permeabilization of the PC-3 cells plasma membrane with digitonin.

In the first description of RH02211, for demonstration of RH02211-induced mtG3PDH inhibition, purified mG3PDH from cDNA of PC-3 cells was incubated with the drug (EC_50_ ± SEM: 28 ± 14 µM) in the presence of 30 mM glycerol-3-P and mtG3PDH activity was assayed by the reduction of Resazurin [23]. In our experimental design with long-term RH02211 exposition of intact PC-3 cells, the significant decrease in DHAP in pyruvate-high RH02211-treated PC-3 cells is consistent with the described inhibition of mtG3PDH. However, in pyruvate-low medium, the observed decrease of glycerol 3-P and increase of DHAP and NAD^+^ rather suggest an activation of mtG3PDH in parallel to an impairment of the cytosolic G3PDH isoenzyme by RH02211 (Fig 5, S1 Table). The decrease in glycerol-3-P levels and an activation of mtG3PDH are metabolically consistent with the measured increase in glycolysis and the increase in H_2_O_2_ production in RH02211-inhibited pyruvate-low PC-3 cells (Figs 1-4, S1 Table). Thus, in human liver tissue, a decrease in glycerol 3-P correlated with an increase in glycolytic flux [54]. Conversely, loss of mtG3PDH activity in the mitochondria-damaged murine carcinoma cell line 4T1 was accompanied by an increase in glycerol-3-P [55]. DHAP is formed together with glyceraldehyde-3-P during the cleavage of fructose 1,6-bisphosphate (Fru-1,6-P2) and is in equilibrium with glyceraldehyde-3-P via the enzyme triosephosphate isomerase. Thus, triose P-isomerase is upregulated in various types of cancer cells, including those of prostate cancer [56].

The IC_50_ value of RH02211 calculated in our study in presence of 2 mM pyruvate (Table 1A) roughly corresponds to the first description of the inhibitor, in which treatment of PC-3 cells with 10 µM RH02211 for 72 hours in presence of 1 mM pyruvate induced an approximately 40% inhibition of cell proliferation [23]. In the same study by Singh (2014), under the same cell culture conditions, the proliferation of the immortalized normal prostate epithelial cell line PNT1A, which contains approximately 17% of mtG3PDH activity compared to PC-3 cells, was inhibited by only 20% in the presence of 10 µM RH02211 [1,23]. Accordingly, our study provides evidence of a correlation between the inhibitory effect of RH02211 on cell division and the ratio between cG3PDH : mtG3PDH of the prostate cancer cell line. In DU145 cells, which were characterized by approximately the same cytosolic G3PDH activity as PC-3 cells but around 40% lower mtG3PDH activity [1], we determined a 3.5-fold higher IC_50_ value for RH02211-induced inhibition of cell proliferation in comparison to PC-3 cells. In contrast to PC-3 cells in DU145 cells 2 mM extracellular pyruvate did not influence the growth inhibitory effect RH02211 (Tables 1A and 1B). The comparison of the enzyme compositions between PC-3 and DU145 cells revealed a higher amount of the M-type of LDH (LDHA) as well as a higher amount of the mitochondrial GOT isoenzyme in PC-3 cells. In contrast, in DU145 cells the H-type of LDH (LDHB) and the cytosolic GOT are dominant (Table 2, S4 and S5 Tables). LDH and GOT both catalyse alternative reactions to the glycerol-3-P shuttle that contribute to the recycling of cytosolic NAD^+^ for the GAPDH reaction. Another difference was found in the impact of extracellular pyruvate on the tetramer : dimer ratio of the pyruvate kinase type M2 that regulates whether glucose carbons are used for glycolytic energy regeneration or channeled in synthetic processes branching from glycolytic intermediates (Table 3) [11]. In addition, Cardoso et al. describe higher glutaminase expression in DU145 cells compared to PC-3 cells, while the expression of the glutamine transporter ASCT2 was not significantly different between the two cell lines [47].

Overall, this study supports the notion that mtG3PDH and the glycerol-3-phosphate shuttle are promising targets for prostate cancer therapy. We also demonstrate that the effects of mtG3PDH inhibitors on both glycolysis and glutaminolysis are dependent on the pyruvate levels, suggesting that caution should be used when testing these drugs and extending the results in vivo.

### Limitations of the study and outlook

The results of the current study are based on the comparison of two AR-negative prostate cancer cell lines with low MPC expression and high dependence on glutamine. Studies on the influence of MCP inhibitors and the role of glutamine metabolism in cell proliferation and metabolism of prostate cancer cells suggest that there are fundamental differences in the metabolic characteristics of AR-positive and AR-negative prostate cancer [42,47]. Therefore, future comparative studies involving AR-positive and castrate-resistant prostate cancer cell lines are needed to determine the role of the metabolic profile of these cells on the antiproliferative effects of mtG3PDH inhibitors.

In our study we considered the impact of pyruvate on the inhibition of cell proliferation by the mtG3PDH inhibitors RH02211 and iGP-1 in monolayer cell cultures. However, in addition to pyruvate, there are a variety of other nutrients, ions, and vitamins whose concentrations differ between commercial culture media and plasma. Cantor et al., 2017 and VandeVoorde et al., 2019 developed media with metabolite concentrations comparable to those of human plasma [46,57]. These media contain 0.05 mM or 0.1 mM pyruvate, respectively, which are in the range of the pyruvate-low medium used in our study with a concentration of 0.02 mM pyruvate. It will be essential to test the effects of these media, in which the concentrations of a wide variety of plasma constituents are adjusted to physiological conditions, on the action of mtG3PDH inhibitors. Still, to fully recapitulate the impact of the microenvironment, the effect of RH02211 should be tested in xenograft models using selected AR-negative and AR-positive cancer cells.

A comparison of steady-state metabolome profiling with hyperpolarized [1-^13^C] pyruvate MRSI in a mouse tumor model using DU145 and PC-3 xenotransplants suggests that steady-state analysis does not adequately assess lactate dehydrogenase activity [58]. To address this aspect in future experiments, either in vitro or in mouse tumor models, flux measurements in the presence of hyperpolarized [1-13C]-pyruvate should be included.

Finally, while here we focused on prostate cancer, a thyroid hormone-dependent increase in mtG3PDH has been described in thyroid cancer, making thyroid cancer another interesting tumor model to investigate the pyruvate-dependent effect of mtG3PDH inhibition on cell proliferation [59].

## Supporting information

S1 Fig Cell density dependencies of metabolite conversion rates in the culture medium supernatants on the example of glutamine consumption in pyruvate-low PC-3 cells. Dependencies were also found for pyruvate production (y = −0.131 ln(x) + 0,50, r = 0.893, n = 18), lactate production (y = −2.976 ln (x) + 15.49, r = 0.879, n = 18) and glutamate production (y = −0.107 ln(x) + 0.46, r = 0.94, n = 18). Cell density dependencies of metabolic conversion rates, intracellular metabolites and enzyme activities are described for MCF-7, MDA-MB-453, transformed rat liver oval cells, NIH 3T3 embryonic mouse fibroblasts and rat hepatocytes (supplementary references 1–4). Accordingly, in this work, cell density dependencies were taken into consideration in the statistical analysis when metabolic conversion rates were compared.
(TIF)

S2 FigDose dependent complete inhibition of cell proliferation in RH02211-treated PC-3 cells cultivated at 21% and 1.5% O_2._A: pyruvate-low medium and B: pyruvate high medium. $\overset{―}{x}$ ± SEM, n ≥ 3.(TIF)

S3 FigReversibility of RH02211 induced inhibition of PC-3 cell proliferation cultivated in pyruvate-low medium.After 96 hours of cultivation in presence of RH02211 as indicated, the medium was replaced by medium without RH02211. After another 96 hours the cells were counted. $\overset{―}{x}$ ± SEM, n = 3. When PC-3 cells were treated for 96 hours with 16 µM RH02211 cell proliferation was completely suppressed. After removal of RH02211 from the medium even at this high concentration cell proliferation started again. However, in the first 96 hours of cultivation in RH02211-free medium cell proliferation resumed slower in comparison to the untreated controls as well as the cells pretreated with 7 µM RH02211.(TIF)

S4 FigDose dependent impact of RH02211 in pyruvate-low PC-3 cells.Due to cell density dependencies (compare S1 Fig) for the statistical comparison, the conversion rates of the different metabolites were adjusted to a global mean cell density (see Statistical analysis). One-way analysis of covariance (ANCOVA) was used to test both the homogeneity of the slopes and the cell density dependencies among the test groups as well as the differences between the adjusted means. A one-way ANOVA (analysis of variance) + Tukey’s test was performed to compare three groups.$\overset{―}{x}$ ± SEM. Control: n ≥ 17; 7 µM RH02211: n = 18; 16 µM RH02211: n ≥ 16, **: p ≤ 0.01.(TIF)

S5 Fig Experimental traces of chemical background from high-resolution respirometry and H_2_O_2_ flux measurements in the absence of sample. (TIF)

S6 FigImpact of pyruvate supplementation on PC-3 cell proliferation in presence of 21% O_2_ (A) and 1.5% O_2_ (B).Pyruvate-high cells were pre-cultivated for 72 hours in pyruvate containing medium. Cell numbers/well after 96 hours of cultivation. $\overset{―}{x}$ ± SEM. White bars = 0.015 mM pyruvate; hatched bars = 2.0 mM pyruvate. Student’s t-test with Mann-Whitney test was performed to assess significancy. *: p ≤ 0.05 and **: p ≤ 0.01. n = 9 (0.015 mM and 2.0 mM pyruvate at 21% O_2_, 2.0 mM pyruvate at 1.5% O_2_), n = 15 (0.015 mM at 1.5% O_2_).(TIF)

S7 FigImpact of extracellular pyruvate on the metabolite conversion rates measured in the culture medium supernatants of PC-3 cells.Due to cell density dependencies (S1 Fig) for the statistical comparison, the conversion rates of the different metabolites were adjusted to a global mean cell density (see Statistical analysis). One-way analysis of covariance (ANCOVA) was used to test both the homogeneity of the slopes and the cell density dependencies among the test groups as well as the differences between the adjusted means. $\overset{―}{x}$± SEM. ***: p ≤ 0.001. n = 18 (0.015 mM pyruvate), n ≥ 17 (2.0 mM pyruvate).(TIF)

S8 Fig Metabolic scheme to the impact of extracellular pyruvate on the metabolic conversion rates measured in the culture medium supernatants as well as on the intracellular concentrations of metabolic intermediates in PC-3 cells. Compare S7 Fig and S3 Table. Extracellular metabolites: bold arrows = increase of the conversion rate; dashed arrows = decrease of the conversion rate. Intracellular metabolites: ↑ = increase of intracellular concentration; ↓ = decrease of intracellular concentration; (--) = concentrations unchanged. In the presence of 2.0 mM pyruvate, the intracellular concentrations of most glycolytic intermediates (glucose, glucose 6-P, fructose 6-P, sum of glycerate 2 and 3-P, lactate) and amino acids (glutamine, aspartate, alanine, serine, 3-P-serine, arginine) increased (S3 Table), which points to an abundance of available metabolic intermediates in pyruvate-high cells. The intracellular concentrations of glutamate itself and proline, which is synthesized from glutamate and the essential amino acids methionine and leucine and semi essential amino acid tyrosine decreased in pyruvate-high cells (S3 Table).
(TIF)

S1 Table Impact of RH02211 on intracellular metabolite concentrations depending upon the pyruvate concentrations in the medium as measured by MS. RH02211 concentration: 7 µM. Independent upon the pyruvate concentration in the medium RH02211 did not affect the concentration of the following intracellular metabolites: Adenosine, S-Adenosyl-homocysteine, S-Adenosyl-methionine, ADP, ADP-ribose AMP, Arginine, ATP, Carbamoyl-aspartate, Carbamoyl-phosphate, CTT, Dihydroorotate, Fumarate, GTP, 2-Hydroxyglutarate, Histidine, Hypoxanthine, 2-Ketoglutarate, Leucine, Lysine, Malate, Methionine, NADH + H^+^, NADP^+^, Phenylalanine, Proline, Sedoheptulose-7-P, Threonine, Tryptophan, Tyrosine, TTP, UDP, UDP-N-acetylglucosamine (UDP-GlcNac), UMP, UTP, Uridine, Valine. Median values with min and max values. ↑ = increase RH02211-treated PC-3 cells; ↓ = decrease in RH02211-treated PC-3 cells; n = 5. Statistical analysis was performed with Wilcoxon Test. The data are shown as median with minimal and maximal values. The significance level was set to p < 0.05.
(PDF)

S2 Table Impact of RH02211 and extracellular pyruvate on the purine: pyrimidine ratio as well as energy charge in PC-3 cells as measured by HPLC. Wilcoxon Test was performed for statistical analysis. The significance level was set to p < 0.05. Mean ± SEM. n = 3. No significances were found between the four groups.
(PDF)

S3 Table Impact of extracellular pyruvate on intracellular metabolites in PC-3 cells measured by MS. Extracellular pyruvate did not affect the concentration of the following intracellular metabolites: Adenosine, ADP-ribose, Asparagine, ATP, Carbamoyl-aspartate, Carbamoyl-phosphate, CTP, DHAP, Dihydroorotate, Fructose-1,6-bisphosphate, Glycine, Glutamylaspartate, GSH, GSSG, GTP, Histidine, Lysine, Methionine, NADH + H^+^, NADP^+^, NADPH + H^+^, Orotate, PEP, Phenylalanine, S-Adenosyl-homocysteine, S-Adenosyl-methionine, Succinate, Threonine, Tryptophan, TTP, UDP, UDP-N-acetylglucosamine (UDP-GlcNac), UDP-glucose, UMP, Uridine, UTP, Valine, XMP. Median values with min and max values. ↑ = increase in pyruvate-high PC-3 cells; ↓ = decrease in pyruvate-high PC-3 cells; n = 5. Statistical analysis was performed with Wilcoxon Test. The data are shown as median with minimal and maximal values. The significance level was set to p < 0.05.
(PDF)

S4 Table Enzymes activities measured in PC-3 (A) and DU145 (B) cells cultivated in pyruvate-low medium. MDHOx = malate dehydrogenase measured in oxaloacetate to malate direction, MDHMa = malate dehydrogenase measured in malate to oxaloacetate direction. Unpaired Student’s t-test was performed to assess significance. Mean ± SEM. n = 3. Neither supplementation of extracellular pyruvate nor RH02211 in concentration of 16 µM had an effect on the enzyme activities of PC-3 cells.
(PDF)

S5 Table Isoelectric points of key metabolic enzymes in PC-3 and DU145 cells cultivated in presence of 0.015 mM pyruvate as measured by isoelectric focusing. MDHOx = malate dehydrogenase measured in oxaloacetate to malate direction; MDHMa = malate dehydrogenase measured in malate to oxaloacetate direction. n = 3. Unpaired Student’s t-test was performed to assess significance. No significant differences between the two cell lines were found. In PC-3 cells neither supplementation of extracellular pyruvate nor 16 µM RH02211 had an impact on the isoelectric points of the listed enzymes.
(PDF)

S1 File Ref Additional references. (PDF)

## Abbreviations

AR: Androgen receptor
CGpDH: Glycerol-3-phosphate dehydrogenase complex
cG3PDH: Cytosolic glycerol-3-P dehydrogenase
DHAP: Dihydroxy acetone phosphate
EN: Enolase
ETS: Electron transfer system
GAPDH: Glyceraldehyde-3-P dehydrogenase
GOT: Glutamate oxaloacetate transaminase
LDH: Lactate dehydrogenase
MDH: Malate dehydrogenase
M2PK, PKM2: Pyruvate kinase isoenzyme m2
mtG3PDH: Mitochondrial glycerol-3-phosphate dehydrogenase
NDPK: Nucleoside diphosphate kinase
PEP: Phosphoenolpyruvate
PFK: 6-phosphofructo-1-kinase
PGK: Phosphoglycerate kinase
PGM: Phosphoglycerate mutase
ROS: Reactive oxygen species
