# Supplementary figures and images for: Impact of mtG3PDH inhibitors on proliferation and metabolism of androgen receptor-negative prostate cancer cells: Role of extracellular pyruvate
Source: PLoS One. 2025 Jun 9;20(6):e0325509. doi: 10.1371/journal.pone.0325509 (PMC12148081; doi:10.1371/journal.pone.0325509)

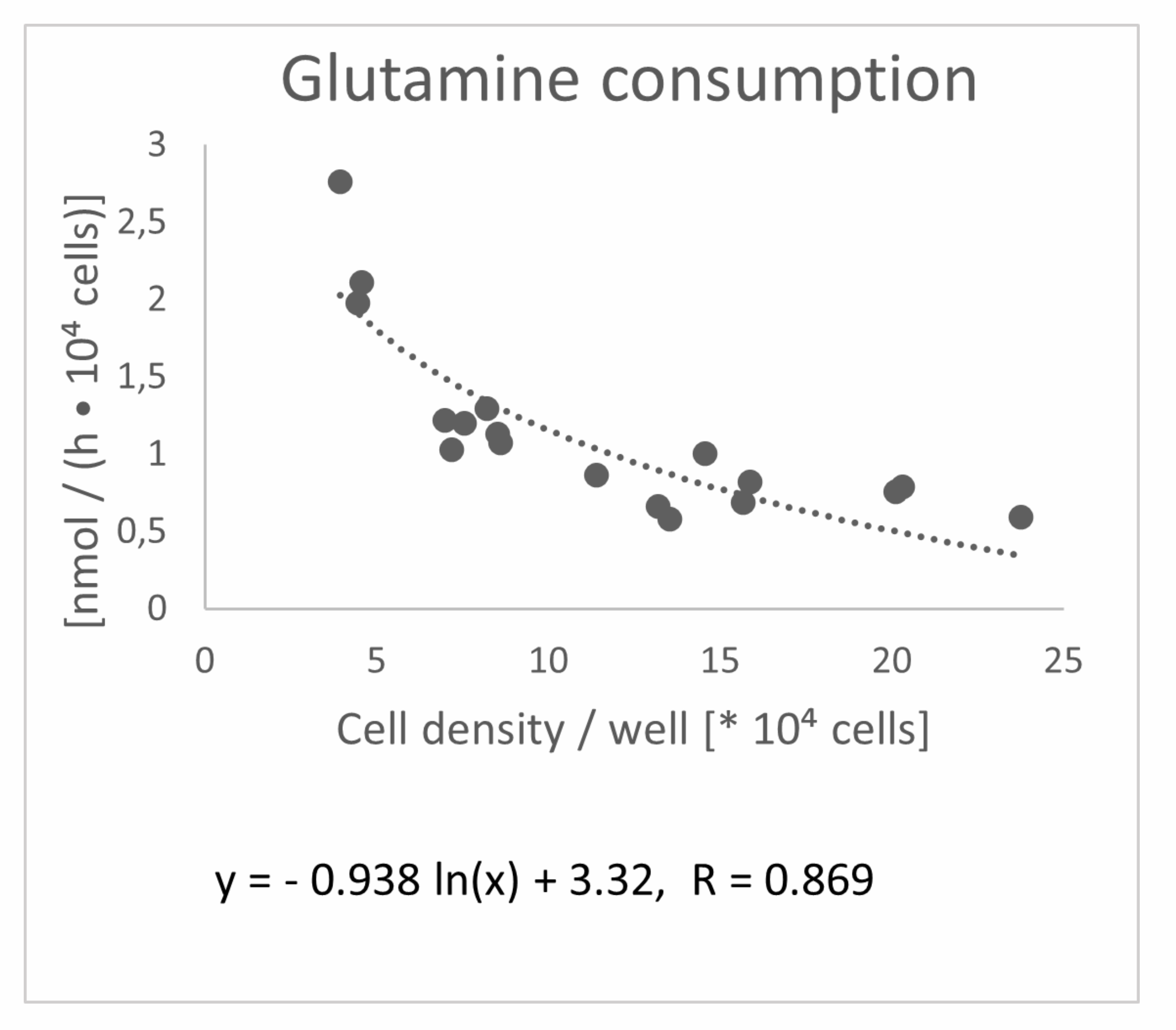

Supplement: S1 Fig — Dependencies were also found for pyruvate production (y = −0.131 ln(x) + 0,50, r = 0.893, n = 18), lactate production (y = −2.976 ln (x) + 15.49, r = 0.879, n = 18) and glutamate production (y = −0.107 ln(x) + 0.46, r = 0.94, n = 18). Cell density dependencies of metabolic conversion rates, intracellular metabolites and enzyme activities are described for MCF-7, MDA-MB-453, transformed rat liver oval cells, NIH 3T3 embryonic mouse fibroblasts and rat hepatocytes (supplementary references 1–4). Accordingly, in this work, cell density dependencies were taken into consideration in the statistical analysis when metabolic conversion rates were compared. (TIF) [file pone.0325509.s001.tif]

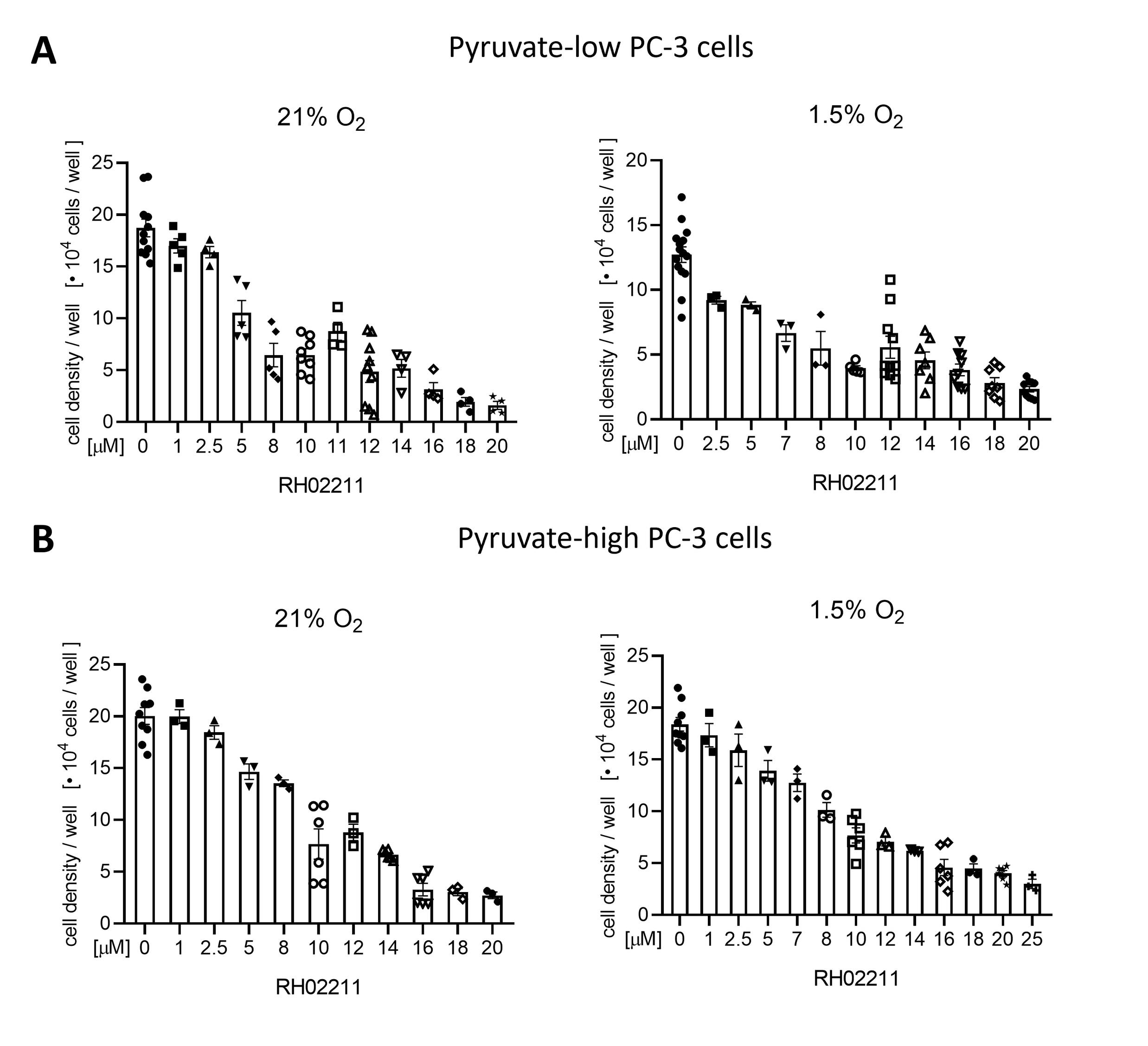

Supplement: S2 Fig — A: pyruvate-low medium and B: pyruvate high medium. x― ± SEM, n ≥ 3. (TIF) [file pone.0325509.s002.tif]

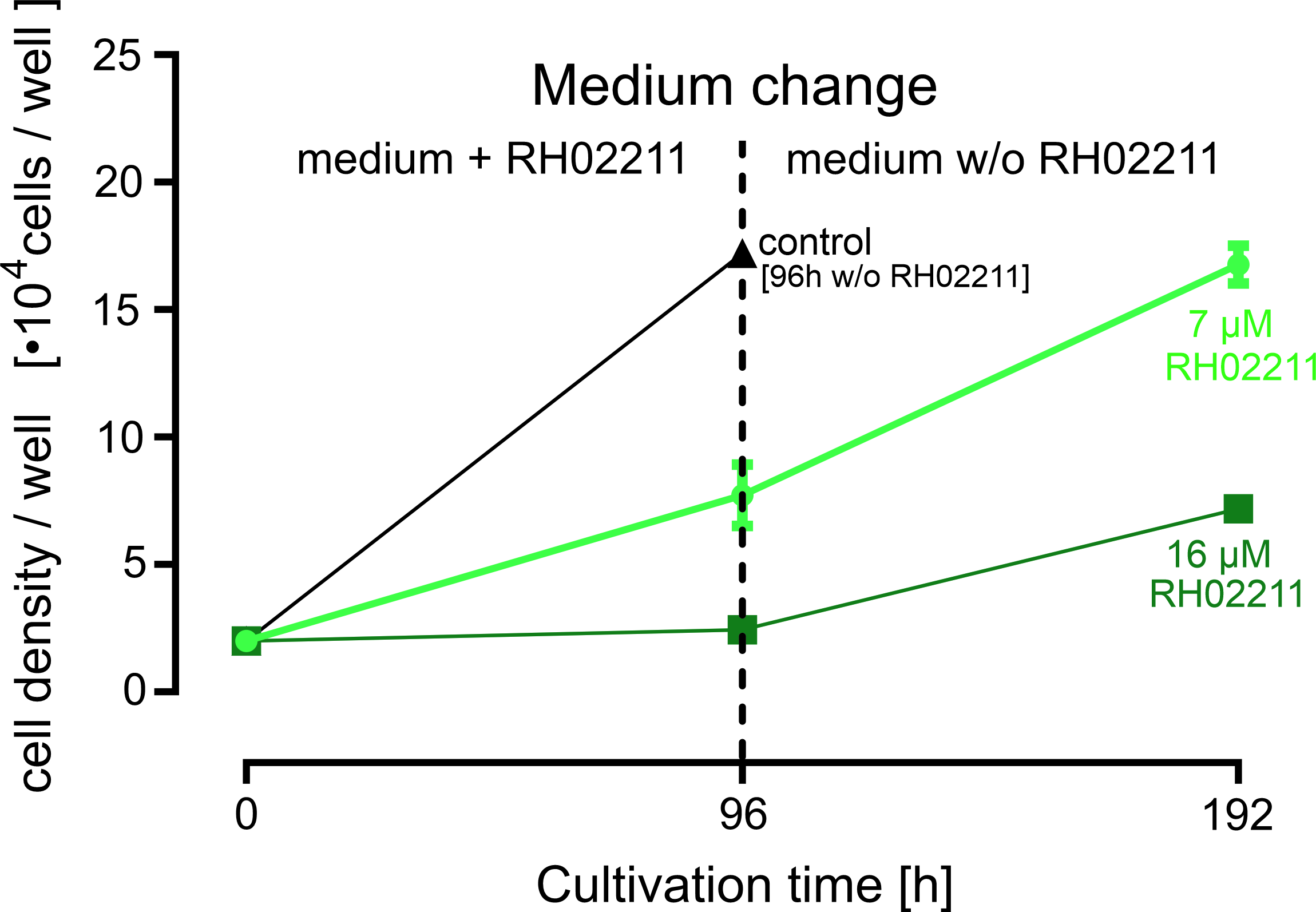

Supplement: S3 Fig — After 96 hours of cultivation in presence of RH02211 as indicated, the medium was replaced by medium without RH02211. After another 96 hours the cells were counted. x― ± SEM, n = 3. When PC-3 cells were treated for 96 hours with 16 µM RH02211 cell proliferation was completely suppressed. After removal of RH02211 from the medium even at this high concentration cell proliferation started again. However, in the first 96 hours of cultivation in RH02211-free medium cell proliferation resumed slower in comparison to the untreated controls as well as the cells pretreated with 7 µM RH02211. (TIF) [file pone.0325509.s003.tif]

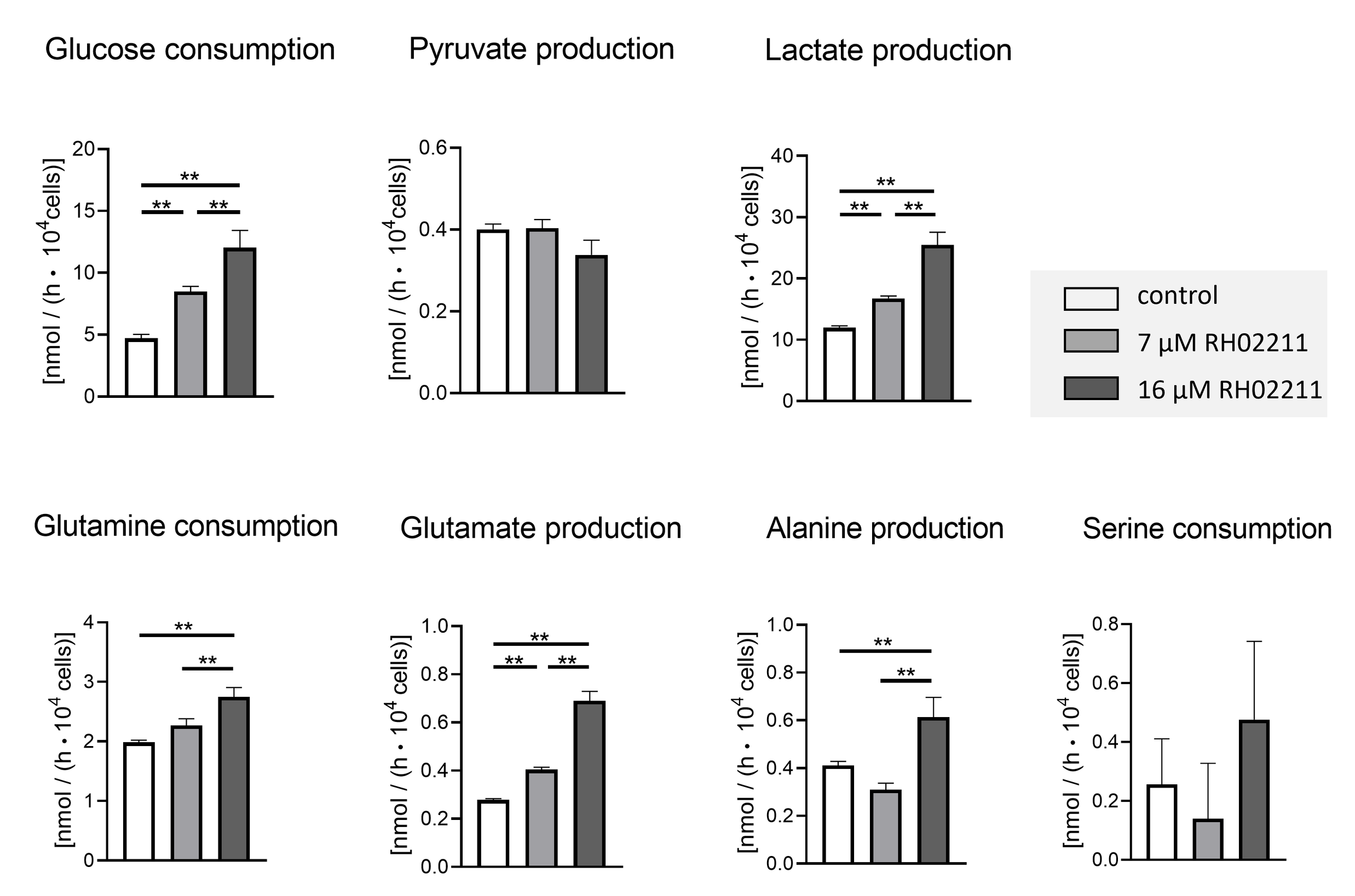

Supplement: S4 Fig — Due to cell density dependencies (compare S1 Fig) for the statistical comparison, the conversion rates of the different metabolites were adjusted to a global mean cell density (see Statistical analysis). One-way analysis of covariance (ANCOVA) was used to test both the homogeneity of the slopes and the cell density dependencies among the test groups as well as the differences between the adjusted means. A one-way ANOVA (analysis of variance) + Tukey’s test was performed to compare three groups.x― ± SEM. Control: n ≥ 17; 7 µM RH02211: n = 18; 16 µM RH02211: n ≥ 16, **: p ≤ 0.01. (TIF) [file pone.0325509.s004.tif]

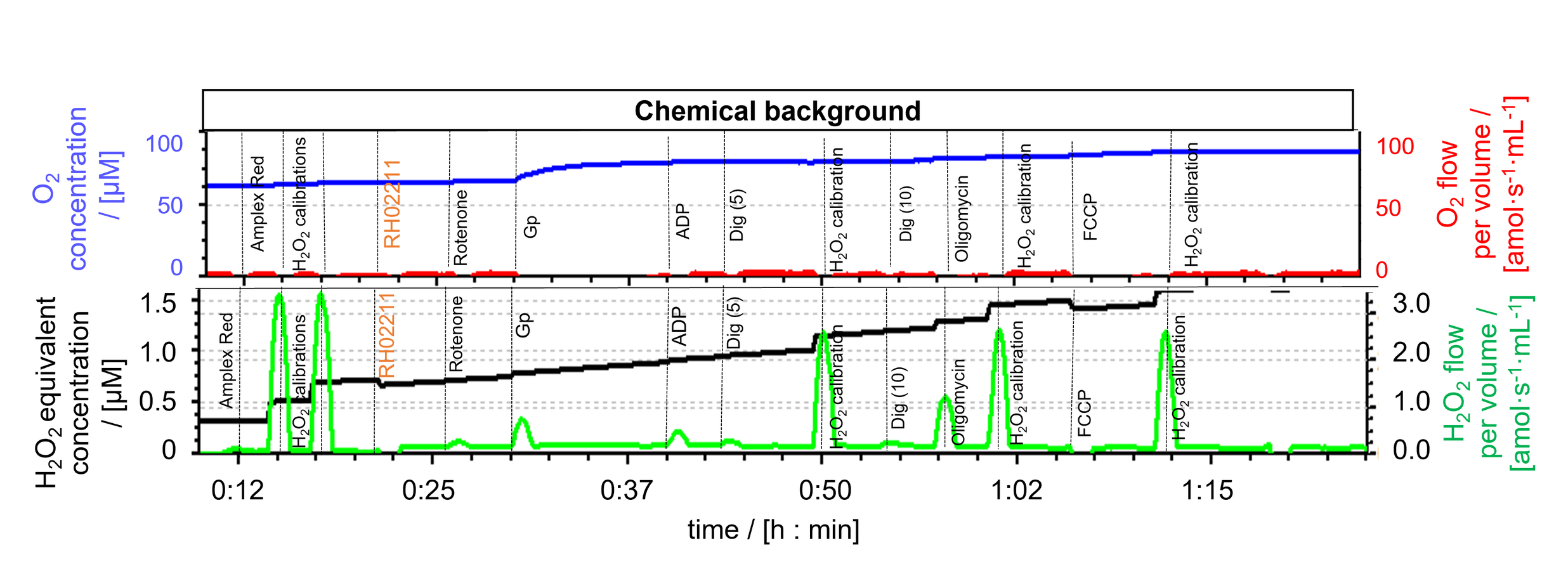

Supplement: S5 Fig — (TIF) [file pone.0325509.s005.tif]

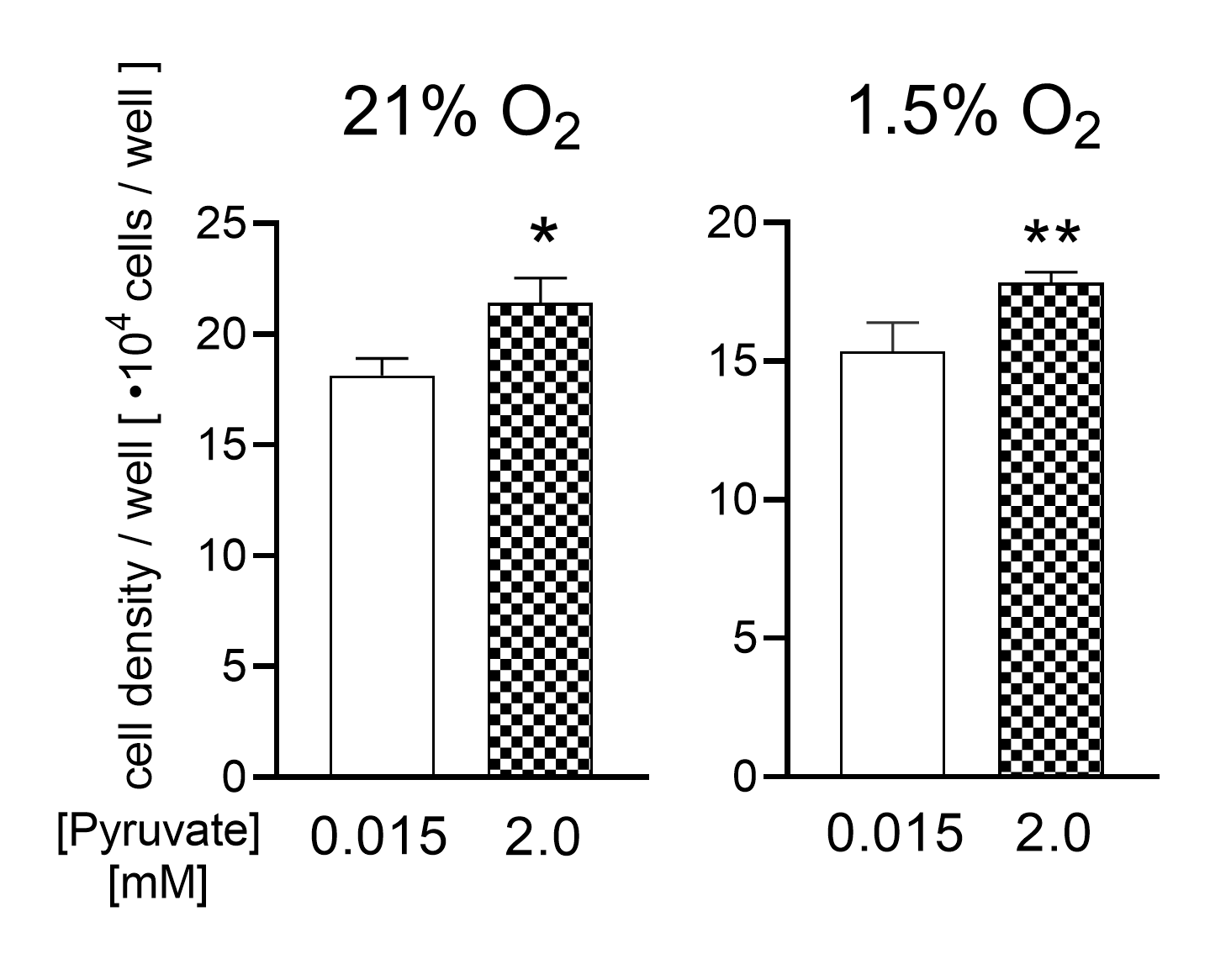

Supplement: S6 Fig — Pyruvate-high cells were pre-cultivated for 72 hours in pyruvate containing medium. Cell numbers/well after 96 hours of cultivation. x― ± SEM. White bars = 0.015 mM pyruvate; hatched bars = 2.0 mM pyruvate. Student’s t-test with Mann-Whitney test was performed to assess significancy. *: p ≤ 0.05 and **: p ≤ 0.01. n = 9 (0.015 mM and 2.0 mM pyruvate at 21% O2, 2.0 mM pyruvate at 1.5% O2), n = 15 (0.015 mM at 1.5% O2). (TIF) [file pone.0325509.s006.tif]

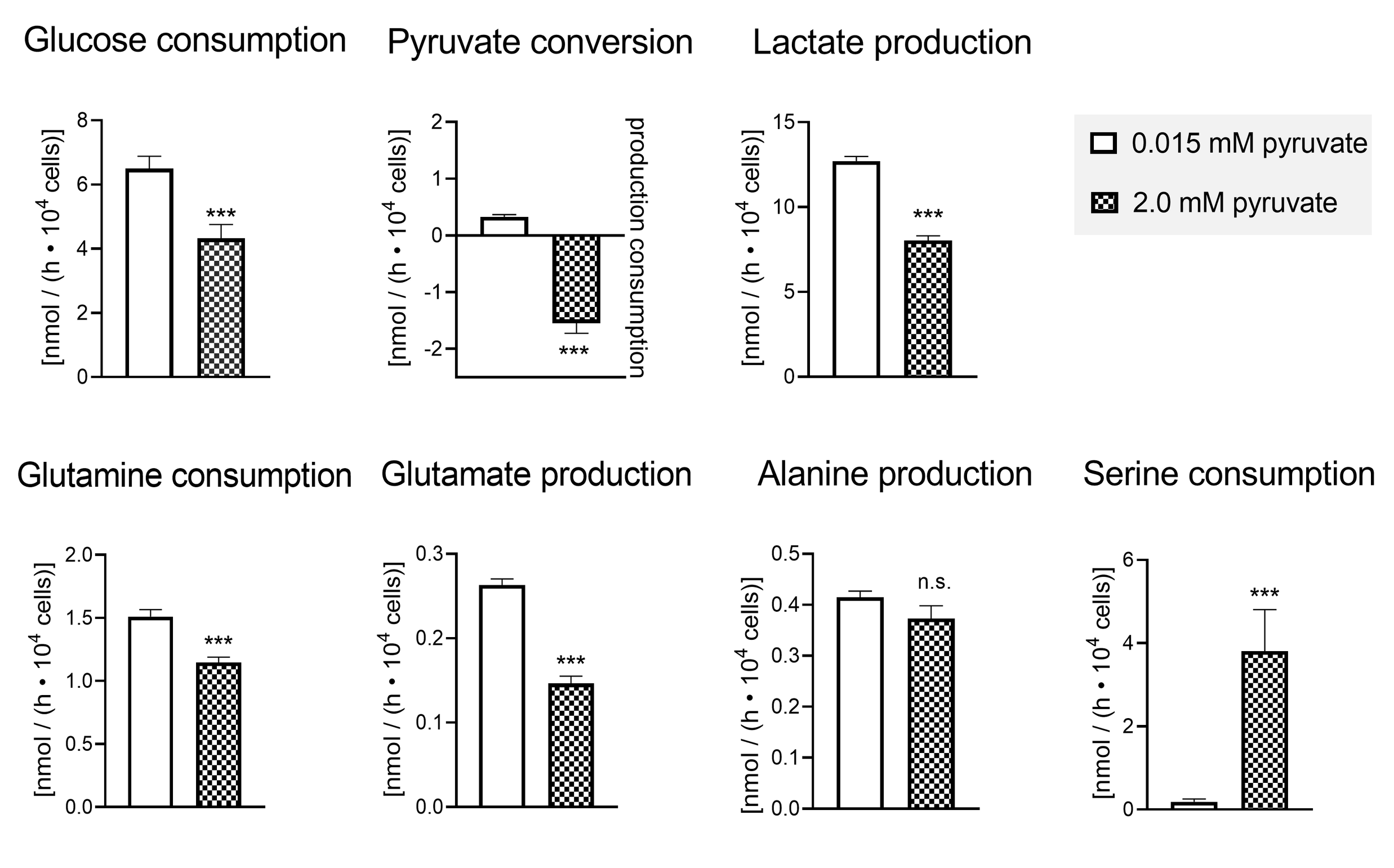

Supplement: S7 Fig — Due to cell density dependencies (S1 Fig) for the statistical comparison, the conversion rates of the different metabolites were adjusted to a global mean cell density (see Statistical analysis). One-way analysis of covariance (ANCOVA) was used to test both the homogeneity of the slopes and the cell density dependencies among the test groups as well as the differences between the adjusted means. x―± SEM. ***: p ≤ 0.001. n = 18 (0.015 mM pyruvate), n ≥ 17 (2.0 mM pyruvate). (TIF) [file pone.0325509.s007.tif]

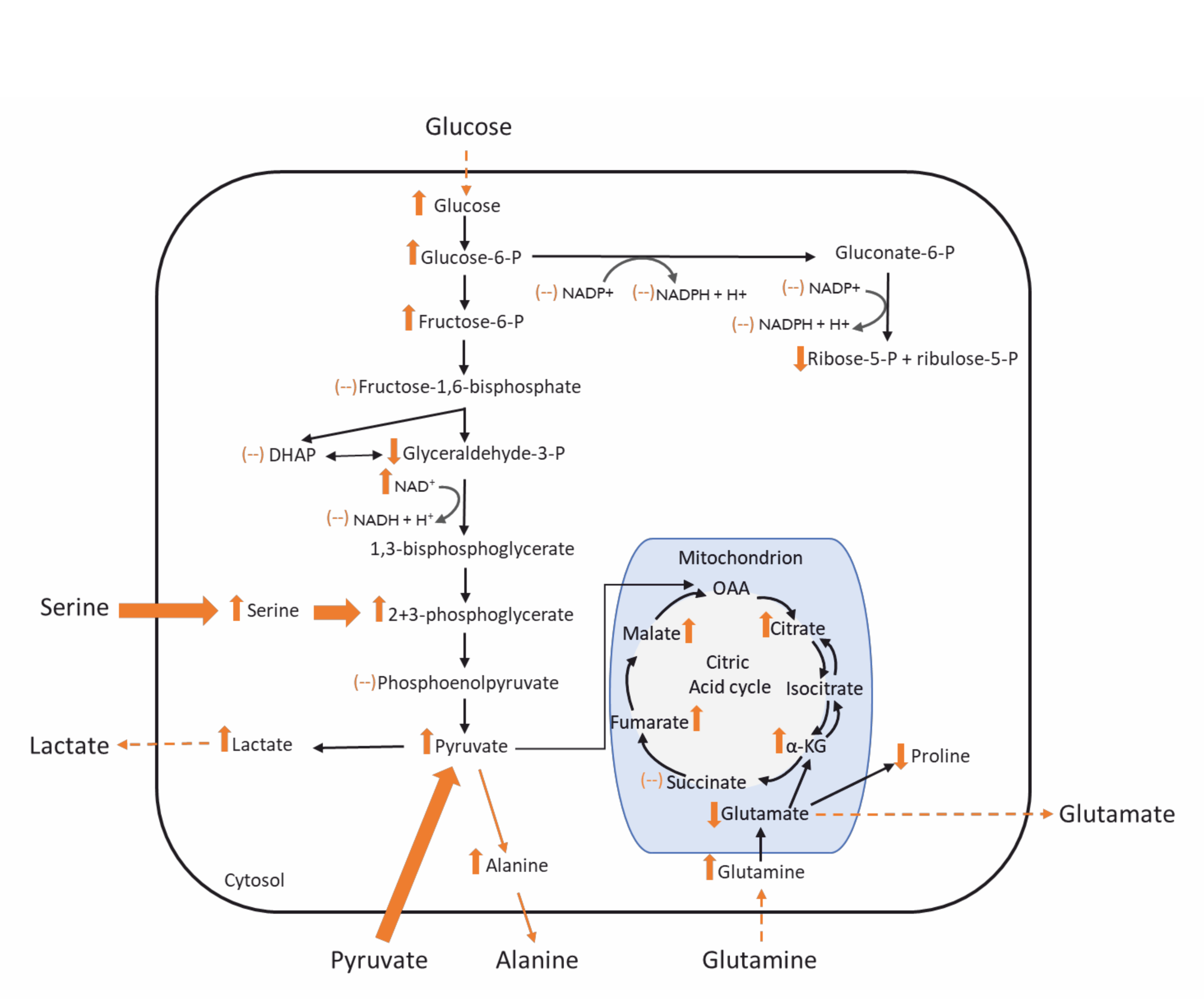

Supplement: S8 Fig — Compare S7 Fig and S3 Table. Extracellular metabolites: bold arrows = increase of the conversion rate; dashed arrows = decrease of the conversion rate. Intracellular metabolites: ↑ = increase of intracellular concentration; ↓ = decrease of intracellular concentration; (--) = concentrations unchanged. In the presence of 2.0 mM pyruvate, the intracellular concentrations of most glycolytic intermediates (glucose, glucose 6-P, fructose 6-P, sum of glycerate 2 and 3-P, lactate) and amino acids (glutamine, aspartate, alanine, serine, 3-P-serine, arginine) increased (S3 Table), which points to an abundance of available metabolic intermediates in pyruvate-high cells. The intracellular concentrations of glutamate itself and proline, which is synthesized from glutamate and the essential amino acids methionine and leucine and semi essential amino acid tyrosine decreased in pyruvate-high cells (S3 Table). (TIF) [file pone.0325509.s008.tif]
