# Supplementary material for: Impact of mtG3PDH inhibitors on proliferation and metabolism of androgen receptor-negative prostate cancer cells: Role of extracellular pyruvate
Source: PLoS One. 2025 Jun 9;20(6):e0325509. doi: 10.1371/journal.pone.0325509 (PMC12148081; doi:10.1371/journal.pone.0325509)
Supplement: S1 Table — Independent upon the pyruvate concentration in the medium RH02211 did not affect the concentration of the following intracellular metabolites: Adenosine, S-Adenosyl-homocysteine, S-Adenosyl-methionine, ADP, ADP-ribose AMP, Arginine, ATP, Carbamoyl-aspartate, Carbamoyl-phosphate, CTT, Dihydroorotate, Fumarate, GTP, 2-Hydroxyglutarate, Histidine, Hypoxanthine, 2-Ketoglutarate, Leucine, Lysine, Malate, Methionine, NADH + H+, NADP+, Phenylalanine, Proline, Sedoheptulose-7-P, Threonine, Tryptophan, Tyrosine, TTP, UDP, UDP-N-acetylglucosamine (UDP-GlcNac), UMP, UTP, Uridine, Valine. Median values with min and max values. ↑ = increase RH02211-treated PC-3 cells; ↓ = decrease in RH02211-treated PC-3 cells; n = 5. Statistical analysis was performed with Wilcoxon Test. The data are shown as median with minimal and maximal values. The significance level was set to p < 0.05. (PDF) [file pone.0325509.s009.pdf]

		0.015 mM Pyruvate			2 mM Pyruvate		
		**Control**	**7 µM RH02211**	**p value**	**Control**	**7 µM RH02211**	**p value**
**Glycolysis and Pentose Phosphate Pathway**	Glucose	7.4 x 10^{7} (min: 7.3 x 10^{7}; max: 7.5 x 10^{7})	8.8 x 10^{7} (min: 8.7 x 10^{7}; max: 11.4 x 10^{7})	0.0122 ↑	10.9 x 10^{7} (min: 9.5 x 10^{7}; max: 13.0 x 10^{7})	9.0 x 10^{7} (min: 8.0 x 10^{7}; max: 12.1 x 10^{7})	n.s
	Glucose-6-P	3.9 x 10^{7} (min: 3.9 x 10^{7}; max: 4.5 x 10^{7})	4.6 x 10^{7} (min: 3.9 x 10^{7}; max: 5.4 x 10^{7})	0.0367 ↑	6.0 x 10^{7} (min: 5.2 x 10^{7}; max: 6.2 x 10^{7})	5.4 x 10^{7} (min: 4.9 x 10^{7}; max: 7.0 x 10^{7})	n.s
	Fructose-6-P	3.6 x 10^{7} (min: 3.5 x 10^{7}; max: 4.0 x 10^{7})	4.3 x 10^{7} (min: 4.0 x 10^{7}; max: 4.5 x 10^{7})	0.0216 ↑	4.1 x 10^{7} (min: 4.0 x 10^{7}; max: 4.5 x 10^{7})	4.3 x 10^{7} (min: 4.0 x 10^{7}; max: 4.7 x 10^{7})	n.s
	Fructose-1,6-bisphosphate	10.0 x 10^{7} (min: 8.6 x 10^{7}; max: 12.9 x 10^{7})	14.9 x 10^{7} (min: 13.9 x 10^{7}; max: 17.3 x 10^{7})	0.0122 ↑	10.3 x 10^{7} (min: 10 x 10^{7}; max: 12.5 x 10^{7})	10.3 x 10^{7} (min: 9.2 x 10^{7}; max: 12.3 x 10^{7})	n.s
	Glyceraldehyde-3-P	1.4 x 10^{10} (min: 1.3 x 10^{10}; max: 1.4 x 10^{10})	1.6 x 10^{10} (min: 1.6 x 10^{10}; max: 1.7 x 10^{10})	0.0122 ↑	1.3 x 10^{10} (min: 1.3 x 10^{10}; max: 1.4 x 10^{10})	1.6 x 10^{10} (min: 1.5 x 10^{10}; max: 1.6 x 10^{10})	0.0122 ↑
	DHAP	11.4 x 10^{7} (min: 9.4 x 10^{7}; max: 12.2 x 10^{7})	13.4 x 10^{7} (min: 12 x 10^{7}; max:15 x 10^{7})	0.0216 ↑	10.3 x 10^{7} (min: 9.8 x 10^{7}; max:11.3 x 10^{7})	9.5 x 10^{7} (min: 8.5 x 10^{7}; max:10 x 10^{7})	0.0216 ↓
	Glycerol-3-P	2.6 x 10^{7} (min: 2.6 x 10^{7}; max: 2.8 x 10^{7})	2.4 x 10^{7} (min: 2.4 x 10^{7}; max: 2.5 x 10^{7})	0.0122 ↓	7.0 x 10^{7} (min:6.4 x 10^{7}; max:8.0 x 10^{7})	7.9 x 10^{7} (min:7.1 x 10^{7}; max:8.4 x 10^{7})	n.s
	2 and 3-phosphoglycerate	9.9 x 10^{7} (min: 9.3 x 10^{7}; max:10.6 x 10^{7})	12.3 x 10^{7} (min: 12.1 x 10^{7}; max: 13.7 x 10^{7})	0.0122 ↑	16.2 x 10^{7} (min: 14.1 x 10^{7}; max:17.3 x 10^{7})	15.0 x 10^{7} (min: 13.4 x 10^{7}; max:16.8 x 10^{7})	n.s
	PEP	1.9 x 10^{9} (min: 1.9 x 10^{9}; max: 2.0 x 10^{9})	1.8 x 10^{9} (min: 1.7 x 10^{9}; max: 1.8 x 10^{9})	0.0122 ↓	2.0 x 10^{9} (min: 1.9 x 10^{9}; max: 2.1 x 10^{9})	1.9 x 10^{9} (min: 1.9 x 10^{9}; max: 2.0 x 10^{9})	n.s
	Pyruvate	1.3 x 10^{7} (min: 1.1 x 10^{7}; max:1.3 x 10^{7})	1.7 x 10^{7} (min: 1.3 x 10^{7}; max: 2 x 10^{7})	0.0367 ↑	7.3 x 10^{7} (min: 5.5 x 10^{7}; max: 9.1 x 10^{7})	5.6 x 10^{7} (min: 4.5 x 10^{7}; max: 8.1 x 10^{7})	n.s
	Lactate	7.6 x 10^{8} (min: 7.2 x 10^{8}; max: 8.3 x 10^{8})	10.6 x 10^{8} (min: 8.7 x 10^{8}; max:12.3 x 10^{8})	0.0122 ↑	11.5 x 10^{8} (min: 9.9 x 10^{8}; max: 12.1 x 10^{8})	10.7 x 10^{8} (min: 9.7 x 10^{8}; max: 12.0 x 10^{8})	n.s
	NAD^{+}	6.4 x 10^{8} (min: 6.4 x 10^{8}; max: 6.5 x 10^{8})	6.1 x 10^{8} (min: 5.9 x 10^{8}; max:6.3 x 10^{8})	0.0122 ↓	6.6 x 10^{8} (min:6.5 x 10^{8}; max:6.8 x 10^{8})	6.5 x 10^{8} (min:6.1 x 10^{8}; max:6.7 x 10^{8})	n.s
	Gluconate	4.8 x 10^{8} (min: 4.5 x 10^{8}; max: 5.4 x 10^{8})	5.5 x 10^{8} (min: 5.1 x 10^{8}; max:6.1 x 10^{8})	0.0216 ↑	6.1 x 10^{8} (min: 6.0 x 10^{8}; max: 6.6 x 10^{8})	7.1 x 10^{8} (min: 6.9 x 10^{8}; max: 10.8 x 10^{8})	0.0122 ↑
	NADPH + H^{+}	9.1 x 10^{6} (min: 7.7 x 10^{6}; max: 11.0 x 10^{6})	11.7 x 10^{6} (min: 10 x 10^{6}; max: 12.6 x 10^{6})	0.0367 ↑	9.5 x 10^{6} (min: 8.6 x 10^{6}; max: 9.9 x 10^{6})	12.1 x 10^{6} (min: 9.3 x 10^{6}; max: 12.9 x 10^{6})	n.s
	Sum of pentose-phosphates	3.5 x 10^{7} (min: 3.4 x 10^{7}; max: 3.7 x 10^{7})	4.0 x 10^{7} (min: 3.8 x 10^{7}; max: 4.2 x 10^{7})	0.0122 ↑	3.0 x 10^{7} (min: 2.9 x 10^{7}; max: 3.2 x 10^{7})	3.2 x 10^{7} (min: 3.1 x 10^{7}; max: 3.3 x 10^{7})	0.0216 ↑
**Citric acid**	Citrate	6.2 x 10^{9} (min: 5.6 x 10^{9}; max: 6.5 x 10^{9})	6.6 x 10^{9} (min: 6.5 x 10^{9}; max: 7.3 x 10^{9})	0.0216 ↑	8.8 x 10^{9} (min: 8.3 x 10^{9}; max: 9.1 x 10^{9})	8.9 x 10^{9} (min: 8.6 x 10^{9}; max: 9.2 x 10^{9})	n.s.
	Cis-Aconitate	1.8 x 10^{8} (min: 1.7 x 10^{8}; max: 1.9 x 10^{8})	2.1 x 10^{8} (min: 2.0 x 10^{8}; max: 2.1 x 10^{8})	0.0122 ↑	3.4 x 10^{8} (min: 3.3 x 10^{8}; max: 3.5 x 10^{8})	3.5 x 10^{8} (min: 3.4 x 10^{8}; max: 3.6 x 10^{8})	n.s.

	Succinate	2.3 x 10^{8} (min: 2.0 x 10^{8}; max: 2.3 x 10^{8})	3.0 x 10^{8} (min: 2.8 x 10^{8}; max: 3.2 x 10^{8})	0.0122 ↑	2.3 x 10^{8} (min: 2.2 x 10^{8}; max: 2.6 x 10^{8})	3.1 x 10^{8} (min: 3.0 x 10^{8}; max: 3.3 x 10^{8})	0.0122 ↑
**Amino acids**	Alanine	7.4 x 10^{8} (min: 7.1 x 10^{8}; max: 7.5 x 10^{8})	7.1 x 10^{8} (min: 7.0 x 10^{8}; max: 7.3 x 10^{8})	n.s.	8.8 x 10^{8} (min: 8.6 x 10^{8}; max: 9.0 x 10^{8})	8.5 x 10^{8} (min: 8.3 x 10^{8}; max: 8.6 x 10^{8})	0.0367 ↓
	Asparagine	6.1 x 10^{7} (min: 5.4 x 10^{7}; max: 6.5 x 10^{7})	5.5 x 10^{7} (min: 4.9 x 10^{7}; max: 5.8 x 10^{7})	n.s.	6.1 x 10^{7} (min: 5.6 x 10^{7}; max: 6.3 x 10^{7})	5.5 x 10^{7} (min: 4.8 x 10^{7}; max: 5.7 x 10^{7})	0.0122 ↓
	Aspartate	1.7 x 10^{9} (min: 1.6 x 10^{9}; max: 1.8 x 10^{9})	1.4 x 10^{9} (min: 1.4 x 10^{9}; max: 1.5 x 10^{9})	0.0122 ↓	1.9 x 10^{9} (min: 1.9 x 10^{9}; max: 2.0 x 10^{9})	1.8 x 10^{9} (min: 1.8 x 10^{9}; max: 1.9 x 10^{9})	0.0216 ↓
	Glycine	3.5 x 10^{7} (min: 3.5 x 10^{7}; max: 3.6 x 10^{7})	4.0 x 10^{7} (min: 3.8 x 10^{7}; max: 4.1 x 10^{7})	0.0122 ↑	2.7 x 10^{7} (min: 2.6 x 10^{7}; max: 2.9 x 10^{7})	3.0 x 10^{7} (min: 2.9 x 10^{7}; max: 3.1 x 10^{7})	0.0216 ↑
	Glutamylaspartate	1.3 x 10^{7} (min: 1.2 x 10^{7}; max: 1.3 x 10^{7})	1.3 x 10^{7} (min: 1.2 x 10^{7}; max: 1.4 x 10^{7}	n.s.	1.2 x 10^{7} (min: 1.2 x 10^{7}; max: 1.3 x 10^{7})	1.4 x 10^{7} (min: 1.4 x 10^{7}; max:1.5 x 10^{7})	0.0122 ↑
	Glutamate	5.0 x 10^{9} (min: 4.9 x 10^{9}; max: 5.0 x 10^{9})	4.5 x 10^{9} (min: 4.4 x 10^{9}; max: 4.6 x 10^{9})	0.0122 ↓	4.4 x 10^{9} (min: 4.3 x 10^{9}; max: 4.4 x 10^{9})	4.3 x 10^{9} (min: 4.3 x 10^{9}; max: 4.4 x 10^{9})	n.s.
	Glutamine	4.0 x 10^{9} (min: 4.0 x 10^{9}; max: 4.2 x 10^{9})	3.9 x 10^{9} (min: 3.7 x 10^{9}; max: 4.0 x 10^{9})	0.0122 ↓	4.3 x 10^{9} (min: 4.3 x 10^{9}; max: 4.4 x 10^{9})	4.0 x 10^{9} (min: 3.9 x 10^{9}; max: 4.1 x 10^{9})	0.0122 ↓
	3-Phosphoserine	2.5 x 10^{6} (min: 2.2 x 10^{6}; max: 3.1 x 10^{6})	2.1 x 10^{6} (min: 2.1 x 10^{6}; max: 2.2 x 10^{6})	0.0367 ↓	9.2 x 10^{6} (min: 9.1 x 10^{6}; max: 9.9 x 10^{6})	8.0 x 10^{6} (min: 7.7 x 10^{6}; max: 8.5 x 10^{6})	0.0122 ↓
	Serine	2.3 x 10^{8} (min: 2.2 x 10^{8}; max: 2.6 x 10^{8})	2.2 x 10^{8} (min: 2.0 x 10^{8}; max: 2.3 x 10^{8})	n.s.	2.6 x 10^{8} (min: 2.6 x 10^{8}; max: 2.8 x 10^{8})	2.5 x 10^{8} (min: 2.4 x 10^{8}; max: 2.5 x 10^{8})	0.0122 ↓
**Purines and Pyrimidines**	ADP	1.6 x 10^{8} (min: 1.5 x 10^{8}; max: 1.7 x 10^{8})	1.5 x 10^{8} (min: 1.4 x 10^{8}; max: 1.7 x 10^{8})	n.s.	1.4 x 10^{8} (min: 1.3 x 10^{8}; max: 1.5 x 10^{8})	1.5 x 10^{8} (min: 1.4 x 10^{8}; max: 1.5 x 10^{8})	0.0367 ↑
	XMP	2.9 x 10^{6} (min: 2.3 x 10^{6}; max: 3.5 x 10^{6})	4.0 x 10^{6} (min: 3.8 x 10^{6}; max: 4.2 x 10^{6})	0.0122 ↑	2.8 x 10^{6} (min: 2.4 x 10^{6}; max: 3.2 x 10^{6})	2.7 x 10^{6} (min: 2.7 x 10^{6}; max: 3.0 x 10^{6})	n.s.
	Uric acid	3.9 x 10^{7} (min: 3.5 x 10^{7}; max: 4.0 x 10^{7})	5.5 x 10^{7} (min: 4.4 x 10^{7}; max: 6.2 x 10^{7})	0.0122 ↑	3.0 x 10^{7} (min: 2.8 x 10^{7}; max: 3.7 x 10^{7})	3.1 x 10^{7} (min: 2.7 x 10^{7}; max: 3.7 x 10^{7})	n.s.
	Orotate	2.3 x 10^{7} (min: 2.2 x 10^{7}; max: 2.7 x 10^{7})	1.5 x 10^{7} (min: 1.4 x 10^{7}; max: 1.7 x 10^{7})	0.0122 ↓	2.1 x 10^{7} (min: 1.9 x 10^{7}; max: 2.3 x 10^{7})	1.9 x 10^{7} (min: 1.7 x 10^{7}; max: 2.1 x 10^{7})	n.s.
	UDP-glucose	6.8 x 10^{8} (min: 6.2 x 10^{8}; max: 6.9 x 10^{8})	6.8 x 10^{8} (min: 6.5 x 10^{8}; max: 7.4 x 10^{8})	n.s.	6.5 x 10^{8} (min: 6.1 x 10^{8}; max: 6.7 x 10^{8})	7.2 x 10^{8} (min: 6.9 x 10^{8}; max: 7.5 x 10^{8})	0.0122 ↑
**Glutat hione**	GSH	1.4 x 10^{10} (min: 1.3 x 10^{10}; max: 1.4 x 10^{10})	1.6 x 10^{10} (min:1.6x10^{10}; max:1.7x10^{10})	0.0122 ↑	1.3 x 10^{10} (min:1.3 x 10^{10}; max: 1.4 x 10^{10})	1.6 x 10^{10} (min:1.5 x 10^{10}; max: 1.6 x 10^{10})	0.0122 ↑
	GSSG	4.4 x 10^{7} (min: 4.2 x 10^{7}; max: 4.7 x 10^{7})	5.9 x 10^{7} (min: 5.6 x 10^{7}; max:6.1 x 10^{7})	0.0122 ↑	4.3 x 10^{7} (min: 3.9 x 10^{7}; max:4.6 x 10^{7})	5.8 x 10^{7} (min: 5.5 x 10^{7}; max: 6.0 x 10^{7})	0.0122 ↑
