# Supplementary material for: Impact of mtG3PDH inhibitors on proliferation and metabolism of androgen receptor-negative prostate cancer cells: Role of extracellular pyruvate
Source: PLoS One. 2025 Jun 9;20(6):e0325509. doi: 10.1371/journal.pone.0325509 (PMC12148081; doi:10.1371/journal.pone.0325509)
Supplement: S2 Table — Wilcoxon Test was performed for statistical analysis. The significance level was set to p < 0.05. Mean ± SEM. n = 3. No significances were found between the four groups. (PDF) [file pone.0325509.s010.pdf]

PC-3 cells	**0.015 mM pyruvate**		**2.0 mM pyruvate**	
	Control	7 µM RH02211	Control	7 µM RH02211
(ATP+GTP) / (UTP+CTP)	1.92 ± 0.07	2.02 ± 0.08	1.91 ± 0.11	1.94 ± 0.08
(ATP+0.5*ADP) / (ATP+ADP+AMP)	0.89 ± 0.02	0.89 ± 0.03	0.87 ± 0.03	0.85 ± 0.03
