# Supplementary material for: Impact of mtG3PDH inhibitors on proliferation and metabolism of androgen receptor-negative prostate cancer cells: Role of extracellular pyruvate
Source: PLoS One. 2025 Jun 9;20(6):e0325509. doi: 10.1371/journal.pone.0325509 (PMC12148081; doi:10.1371/journal.pone.0325509)
Supplement: S3 Table — Median values with min and max values. ↑ = increase in pyruvate-high PC-3 cells; ↓ = decrease in pyruvate-high PC-3 cells; n = 5. Statistical analysis was performed with Wilcoxon Test. The data are shown as median with minimal and maximal values. The significance level was set to p < 0.05. (PDF) [file pone.0325509.s011.pdf]

		PC-3 cells		
		0.015 mM Pyruvate	2 mM Pyruvate	p value
Glycolysis and Pentose Phosphate Pathway	Glucose	7.4×10^7 (min: 7.3×10^7; max: 7.5×10^7)	10.9×10^7 (min: 9.5×10^7; max: 13.0×10^7)	0.0122 ↑
	Glucose-6-P	4.0×10^7 (min: 3.9×10^7; max: 4.5×10^7)	6.0×10^7 (min: 5.2×10^7; max: 6.2×10^7)	0.0122 ↑
	Fructose-6-P	3.6×10^7 (min: 3.5×10^7; max: 4.0×10^7)	4.1×10^7 (min: 4.0×10^7; max: 4.5×10^7)	0.0367 ↑
	Glyceraldehyde-3-P	1.4×10^{10} (min: 1.3×10^{10}; max: 1.4×10^{10})	1.3×10^{10} (min: 1.3×10^{10}; max: 1.4×10^{10})	0.0122 ↓
	Glycerol-3-P	2.6×10^7 (min: 2.6×10^7; max: 2.8×10^7)	7.0×10^7 (min: 6.4×10^7; max: 8.0×10^7)	0.0122 ↑
	2 and 3-phosphoglycerate	9.9×10^7 (min: 9.3×10^8; max: 1.1×10^8)	16.2×10^7 (min: 1.4×10^8; max: 1.7×10^8)	0.0122 ↑
	Pyruvate	1.3×10^7 (min: 1.1×10^7; max: 1.3×10^7)	7.3×10^7 (min: 5.5×10^7; max: 9.1×10^7)	0.0122 ↑
	Lactate	7.6×10^8 (min: 7.2×10^8; max: 8.3×10^8)	11.5×10^8 (min: 9.9×10^8; max: 12.1×10^8)	0.0122 ↑
	NAD^+	6.4×10^8 (min: 6.4×10^8; max: 6.5×10^8)	6.6×10^8 (min: 6.5×10^8; max: 6.8×10^8)	0.0122 ↑
	Gluconate	4.8×10^8 (min: 4.5×10^8; max: 5.4×10^8)	6.1×10^8 (min: 6.0×10^8; max: 6.6×10^8)	0.0122 ↑
	Sum of pentose-phosphates	3.5×10^7 (min: 3.4×10^7; max: 3.7×10^7)	3.0×10^7 (min: 2.9×10^7; max: 3.2×10^7)	0.0122 ↓
	Sedoheptulose-7-P	1.4×10^7 (min: 1.4×10^7; max: 1.6×10^7)	1.2×10^7 (min: 1.1×10^7; max: 1.3×10^7)	0.0122 ↓
Citric acid cycle	Citrate	6.2×10^9 (min: 5.6×10^9; max: 6.5×10^9)	8.8×10^9 (min: 8.3×10^9; max: 9.1×10^9)	0.0122 ↑
	Cis-aconitate	1.8×10^8 (min: 1.7×10^8; max: 1.9×10^8)	3.4×10^8 (min: 3.3×10^8; max: 3.5×10^8)	0.0122 ↑
	2-Ketoglutarate	2.2×10^8 (min: 2.2×10^8; max: 2.3×10^8)	6.4×10^8 (min: 6.0×10^8; max: 6.7×10^8)	0.0122 ↑
	2-Hydroxyglutarate	9.6×10^7 (min: 8.4×10^7; max: 9.8×10^7)	1.4×10^7 (min: 1.3×10^7; max: 1.4×10^7)	0.0122 ↓
	Fumarate	2.6×10^8 (min: 2.3×10^8; max: 2.6×10^8)	3.0×10^8 (min: 2.9×10^8; max: 3.2×10^8)	0.0122 ↑
	Malate	3.3×10^9 (min: 3.0×10^9; max: 3.4×10^9)	3.9×10^9 (min: 3.6×10^9; max: 4.1×10^9)	0.0122 ↑
Amino acids	Alanine	7.4×10^8 (min: 7.1×10^8; max: 7.5×10^8)	8.8×10^8 (min: 8.6×10^8; max: 9.0×10^8)	0.0122 ↑
	Arginine	2.1×10^9 (min: 2.0×10^9; max: 2.2×10^9)	2.5×10^9 (min: 2.1×10^9; max: 2.8×10^9)	0.0367 ↑
	Aspartate	1.7×10^9 (min: 1.6×10^9; max: 1.8×10^9)	1.9×10^9 (min: 1.9×10^9; max: 2.0×10^9)	0.0122 ↑
	Cysteic acid	1.7×10^6 (min: 1.7×10^6; max: 1.8×10^6)	1.6×10^6 (min: 1.5×10^6; max: 1.7×10^6)	0.0122 ↓
	Glutamate	4.9×10^9 (min: 4.9×10^9; max: 5.0×10^9)	4.4×10^9 (min: 4.3×10^9; max: 4.4×10^9)	0.0122 ↓

	Glutamine	4.0×10^9 (min: 4.0×10^9; max: 4.2×10^9)	4.4×10^9 (min: 4.3×10^9; max: 4.4×10^9)	0.0122 ↑
	Leucine	2.0×10^{10} (min: 1.9×10^{10}; max: 2.0×10^{10})	1.9×10^{10} (min: 1.8×10^{10}; max: 1.9×10^{10})	0.0367 ↓
	Methionine	6.0×10^9 (min: 5.9×10^9; max: 6.3×10^9)	5.7×10^9 (min: 5.6×10^9; max: 5.9×10^9)	0.0122 ↓
	Proline	1.9×10^{10} (min: 1.8×10^{10}; max: 1.9×10^{10})	1.7×10^{10} (min: 1.6×10^{10}; max: 1.7×10^{10})	0.0122 ↓
	Serine	2.3×10^8 (min: 2.2×10^8; max: 2.6×10^8)	2.6×10^8 (min: 2.6×10^8; max: 2.8×10^8)	0.0122 ↑
	3-Phosphoserine	2.5×10^6 (min: 2.2×10^6; max: 3.1×10^6)	9.2×10^6 (min: 9.1×10^6; max: 9.9×10^6)	0.0122 ↑
	Tyrosine	6.4×10^9 (min: 6.3×10^9; max: 6.9×10^9)	6.1×10^9 (min: 5.9×10^9; max: 6.3×10^9)	0.0122 ↓
**Purines and Pyrimidines**	AMP	2.9×10^7 (min: 2.7×10^7; max: 3.0×10^7)	2.3×10^7 (min: 2.2×10^7; max: 2.8×10^7)	0.0367 ↓
	ADP	1.6×10^8 (min: 1.5×10^8; max: 1.7×10^8)	1.4×10^8 (min: 1.3×10^8; max: 1.5×10^8)	0.0122 ↓
	Hypoxanthine	6.5×10^6 (min: 5.6×10^6; max: 8.8×10^6)	5.3×10^6 (min: 3.4×10^6; max: 5.7×10^6)	0.0367 ↓
	Uric acid	3.9×10^7 (min: 3.5×10^7; max: 4.0×10^7)	3.0×10^7 (min: 2.8×10^7; max: 3.7×10^7)	0.0367 ↓
