# Supplementary material for: Impact of mtG3PDH inhibitors on proliferation and metabolism of androgen receptor-negative prostate cancer cells: Role of extracellular pyruvate
Source: PLoS One. 2025 Jun 9;20(6):e0325509. doi: 10.1371/journal.pone.0325509 (PMC12148081; doi:10.1371/journal.pone.0325509)
Supplement: S4 Table — MDHOx = malate dehydrogenase measured in oxaloacetate to malate direction, MDHMa = malate dehydrogenase measured in malate to oxaloacetate direction. Unpaired Student’s t-test was performed to assess significance. Mean ± SEM. n = 3. Neither supplementation of extracellular pyruvate nor RH02211 in concentration of 16 µM had an effect on the enzyme activities of PC-3 cells. (PDF) [file pone.0325509.s012.pdf]

[U/10^7 cells]	PC-3 cells		DU145 cells		Significance
	$\bar{x}$	*SEM*	$\bar{x}$	*SEM*	p value (PC-3 vs DU145)
Aldolase	0.67	0.06	1.28	0.33	n.s.
GAPDH	7.48	1.28	9.96	2.47	n.s.
PGK	3.61	0.30	1.73	0.43	0.0238
PGM	1.88	0.21	2.11	0.40	n.s.
PK	5.37	1.59	6.48	1.54	n.s.
LDH	18.37	0.66	11.82	2.06	0.0388
NDPK	4.13	0.24	2.55	0.41	0.0290
MDHOx	4.58	1.50	3.35	0.65	n.s.
MDHMa	0.24	0.08	0.26	0.14	n.s.
GOT	0.53	0.09	0.67	0.13	n.s.
