# Supplementary material for: Impact of mtG3PDH inhibitors on proliferation and metabolism of androgen receptor-negative prostate cancer cells: Role of extracellular pyruvate
Source: PLoS One. 2025 Jun 9;20(6):e0325509. doi: 10.1371/journal.pone.0325509 (PMC12148081; doi:10.1371/journal.pone.0325509)
Supplement: S5 Table — MDHOx = malate dehydrogenase measured in oxaloacetate to malate direction; MDHMa = malate dehydrogenase measured in malate to oxaloacetate direction. n = 3. Unpaired Student’s t-test was performed to assess significance. No significant differences between the two cell lines were found. In PC-3 cells neither supplementation of extracellular pyruvate nor 16 µM RH02211 had an impact on the isoelectric points of the listed enzymes. (PDF) [file pone.0325509.s013.pdf]

IEP	**PC-3 cells**		**DU145 cells**	
	$\bar{x}$	SEM	$\bar{x}$	SEM
Aldolase	8.0	0.1	7.8	0.2
GAPDH	6.5	0.1	6.5	0.3
PGK	7.3	0.0	6.9	0.1
PGM	5.4	0.4	5.2	0.2
PK	6.9	0.1	6.7	0.2
LDH - H4	5.4	0.2	5.2	0.1
LDH - Hybrids	6.5	0.1	6.3	0.2
LDH - M4	8.7	0.1	8.5	0.1
NDPK	6.2	0.0	5.7	0.3
	7.1	0.1	6.6	0.2
	8.5	0.0	8.1	0.2
MDHOx cyto.	5.4	0.2	5.2	0.1
prec.	7.8	0.0	7.6	0.2
mito.	8.8	0.1	8.7	0.0
MDHMa cyto.	5.4	0.2	5.3	0.1
prec.	0.0	0.0	0.0	0.0
mito.	8.8	0.3	8.6	0.0
GOT cyto	5.8	0.1	5.3	0.1
mito.	9.0	0.0	8.7	0.0
