# Supplementary material for: Impact of mtG3PDH inhibitors on proliferation and metabolism of androgen receptor-negative prostate cancer cells: Role of extracellular pyruvate
Source: PLoS One. 2025 Jun 9;20(6):e0325509. doi: 10.1371/journal.pone.0325509 (PMC12148081; doi:10.1371/journal.pone.0325509)
Supplement: S1 File Ref — (PDF) [file pone.0325509.s014.pdf]

References

1. Jurin RR, McCune SA. Effect of cell density on metabolism in isolated rat hepatocytes. J Cell Physiol. 1985 Jun;123(3):442–8.

2. Mazurek S, Michel A, Eigenbrodt E. Effect of extracellular AMP on cell proliferation and metabolism of breast cancer cell lines with high and low glycolytic rates. J Biol Chem. 1997;272(8):4941–52.

3. Mazurek S, Eigenbrodt E, Failing K, Steinberg P. Alterations in the glycolytic and glutaminolytic pathways after malignant transformation of rat liver oval cells. J Cell Physiol. 1999;181(1):136–46.

4. Mazurek S, Zwerschke W, Jansen-Dürr P, Eigenbrodt E. Effects of the human papilloma virus HPV-16 E7 oncoprotein on glycolysis and glutaminolysis: Role of pyruvate kinase type M2 and the glycolytic-enzyme complex. Biochem J. 2001;356(1):247–56.
